# Genome-wide RNAi Screen Reveals a New Role of a WNT/CTNNB1 Signaling Pathway as Negative Regulator of Virus-induced Innate Immune Responses

**DOI:** 10.1371/journal.ppat.1003416

**Published:** 2013-06-13

**Authors:** Martin Baril, Salwa Es-Saad, Laurent Chatel-Chaix, Karin Fink, Tram Pham, Valérie-Ann Raymond, Karine Audette, Anne-Sophie Guenier, Jean Duchaine, Marc Servant, Marc Bilodeau, Éric Cohen, Nathalie Grandvaux, Daniel Lamarre

**Affiliations:** 1 Institut de Recherche en Immunologie et en Cancérologie (IRIC), Université de Montréal, Montréal, Québec, Canada; 2 Centre de Recherche du CHUM (CRCHUM), Hôpital Saint-Luc, Montréal, Québec, Canada; 3 Institut de Recherches Cliniques de Montréal (IRCM), Montréal, Québec, Canada; 4 Faculté de Pharmacie, Université de Montréal, Montréal, Québec, Canada; 5 Faculté de Médecine, Université de Montréal, Montréal, Québec, Canada; Harvard Medical School, United States of America

## Abstract

To identify new regulators of antiviral innate immunity, we completed the first genome-wide gene silencing screen assessing the transcriptional response at the interferon-β (*IFNB1*) promoter following Sendai virus (SeV) infection. We now report a novel link between WNT signaling pathway and the modulation of retinoic acid-inducible gene I (RIG-I)-like receptor (RLR)-dependent innate immune responses. Here we show that secretion of WNT2B and WNT9B and stabilization of β-catenin (CTNNB1) upon virus infection negatively regulate expression of representative inducible genes *IFNB1*, *IFIT1* and *TNF* in a CTNNB1-dependent effector mechanism. The antiviral response is drastically reduced by glycogen synthase kinase 3 (GSK3) inhibitors but restored in CTNNB1 knockdown cells. The findings confirm a novel regulation of antiviral innate immunity by a canonical-like WNT/CTNNB1 signaling pathway. The study identifies novel avenues for broad-spectrum antiviral targets and preventing immune-mediated diseases upon viral infection.

## Author Summary

The innate immune system is the first line of defense for organisms that possess an adaptive immune system. It allows a rapid immune response upon viral infections, in addition to propagating an antiviral state in neighboring cells. In an attempt to identify new proteins that are involved in antiviral responses, we completed the first genome-wide RNA interference (RNAi) screen by individually silencing the expression of 15,000 human genes to assess their role in the induction of type I interferon-β (*IFNB1*) upon Sendai virus (SeV) infection. We identified 237 potential modulator genes for which negative or positive actions of gene products were mapped to the different steps of the antiviral responses. Among these genes, we find two members of the WNT family WNT2B and WNT9B that negatively regulate the *IFNB1* response. We show that SeV infection induces the secretion of WNT ligands, stabilization of β-catenin (CTNNB1) and decreases expression of IFNB1 in a feedback mechanism. We further demonstrate that inhibition of glycogen synthase kinase 3 (GSK3) reduces the antiviral innate response in a CTNNB1-dependent manner. The findings suggest that drugs targeting this newly identified canonical-like WNT/CTNNB1 signaling pathway may be therapeutically useful to modulate viral replication or virus-induced inflammation.

## Introduction

The innate immune system is the first line of defense for organisms that possess an adaptive immune system. It relies on the presence of specific pattern recognition receptors (PRRs) that recognize pathogen-associated molecular patterns (PAMPs) to induce expression of cytokines such as type I interferons (IFNs), pro-inflammatory cytokines and chemokines. Through the induction of IFN-stimulated genes (ISGs) upon viral infection, type I IFNs are critical components of the innate immune response in virtually all cells and the target of viral immune evasion strategies. Upon viral infection, recognition of foreign nucleic acids is made through extracellular sensing by endosomal Toll-Like Receptors (TLRs 3, 7, 8 and 9) or intracellular detection by specific DExD-box RNA helicases: retinoic acid-inducible gene I (RIG-I also known as DDX58), melanoma differentiation–associated gene-5 (MDA5, also known as IFIH1) and laboratory of genetics and physiology 2 (LGP2, also known as DHX58), which form the RIG-I-like receptors (RLRs) family [1]. In response to viral infection, these RLRs associate with the mitochondrial antiviral signaling (MAVS) adaptor (also named IPS-1, Cardif and VISA) [2]–[5], leading to the activation of key transcriptional factors such as interferon regulatory factor 3 (IRF3) and nuclear factor of kappa light polypeptide gene enhancer in B-cells (NF-κB), induction of type I IFN, and ultimately production of hundreds of ISGs. This antiviral effector program is a fundamental target for virus-encoded immune suppression [6]. An outstanding example is the hepatitis C virus (HCV) NS3/4A protease that cleaves TRIF and MAVS adaptor molecules in the TLR3 and RLR pathways, respectively, to block IRF3-dependent expression of IFNB1 and IFN-mediated cellular antiviral response [7]. Therefore, regulation of *IFNB1* gene expression is important for efficient antiviral response, but is also required to prevent negative effects of an extended duration of type I IFN production [8]. Two previous RNAi screens focused on regulation of innate immunity, but were both performed in drosophila following bacterial infection [9], [10]. In an effort to identify regulators of the innate antiviral response in human, we completed the first genome-wide RNAi screen assessing SeV-induced *IFNB1* transcription in embryonic kidney HEK 293T cells. We identified 237 potential modulator genes for which negative or positive actions of gene products were mapped to the different steps of the antiviral responses from virus sensing, signal propagation/amplification up to the feedback regulation. In the present study, we described specific WNT ligands activating a canonical-like WNT/CTNNB1 pathway as a previously unrecognized effector mechanism to negatively regulate antiviral innate immunity.

## Results

### Genome-wide RNAi screen identify modulators of SeV-induced *IFNB1* expression

**Figure 1:**
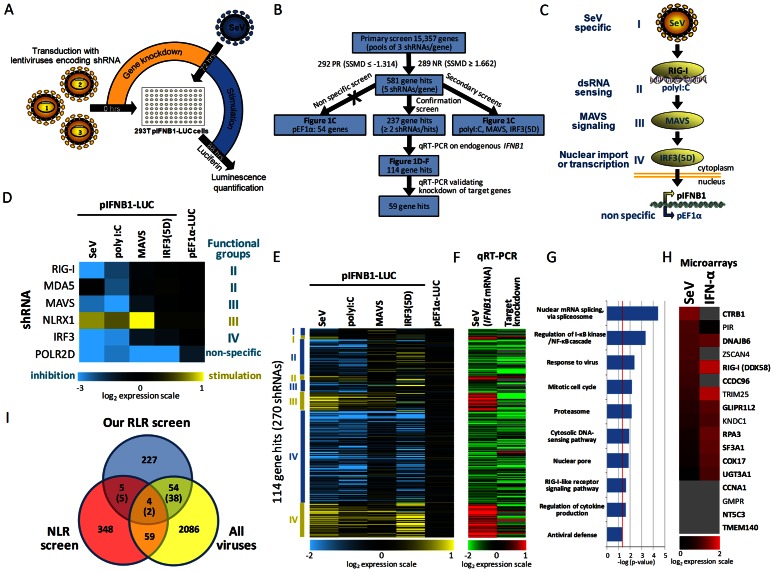
Genome-wide gene silencing study of virus-induced innate immune responses and bioinformatics analyses. (A) Schematic representation of the primary genome-wide screen and secondary screens. HEK 293T cells stably expressing the luciferase gene under the control of the *IFNB1* promoter were transduced with arrayed lentiviruses combining three shRNAs per gene (primary screen) or with five individual shRNA-expressing lentiviruses for each gene hit (secondary screens) in a 96-well format. After 72 hours, cells were challenged with SeV virus (primary and confirmation screens) or transfected with polyinosinic∶polycytidylic acid (polyI∶C), MAVS- or IRF3(5D)-expressing plasmids (secondary screens) for 16 hours before measuring *IFNB1* promoter-driven luciferase activity. (B) Decision tree of primary screen and summary data of gene hits obtained in secondary and validation screens. Selected gene hits (114) that were confirmed and validated with endogenous *IFNB1* screens by qRT-PCR induced a modulation of more than 25% of the *IFNB1* promoter activity with at least two independent shRNAs following SeV infection. Prioritized gene hits (59) for which knockdown of the target gene was greater than 40% with two independent shRNAs are also identified. (C) Schematic representation of confirmation and secondary assays for epistasis analysis of gene hits acting on the signaling cascade leading to *IFNB1* production. SeV infection (primary and confirmation screens), polyI∶C (dsRNA mimetic), MAVS or IRF3(5D) expressing plasmids transfection (secondary screens) were used to activate innate immune response. A non-specific assay was used to discard gene hits affecting nonimmune-related transcription by measuring transcriptional activity of EF1α constitutive promoter. (D) Heat map indicating modulation of *IFNB1* promoter activity following silencing of control genes in confirmation and secondary assays (log_2_ scale). The functional profiling data for controls and gene hits allow classification within four functional groups: I - SeV specific, II - cytoplasmic dsRNA sensing, III - MAVS-dependent signaling, IV - nuclear import or transcription factor-dependent process. (E) Functional profiling data of 114 gene hits confirmed with at least two shRNAs in the SeV confirmation screen and further validated using endogenous *IFNB1* mRNA quantification by qRT-PCR. Manual clustering was performed to classify each gene hit in one of the four functional groups. (F) qRT-PCR validation data of the endogenous *IFNB1* mRNA levels and target gene knockdown efficiency in transduced cells with each lentivirus-expressing shRNA for the 114 gene hits. (G) Enriched Gene Ontology (GO) biological process and molecular function terms (P<0.05) for the 114 genes confirmed by qRT-PCR relative to all genes examined in the genome-wide RNAi screen. (H) Identification of 17 out of 237 gene hits that are induced by more than twofold after SeV infection or IFN-α treatment in our microarray analysis of HEK 293T cells (13 genes) or previously described as ISGs (4 genes in gray) [75]. The gene hits that modulate endogenous *IFNB1* mRNA are indicated in bold. (I) Venn diagram representing the overlap between various RNAi screens including: our RLR RNAi screen (290 genes validated in secondary screens, including 53 hits considered as non specific in pEF1α assay), a NOD-like receptor screen (NLR) and nine independent viral replication screens (HIV, influenza, HCV and West Nile virus). Numbers between brackets represent the overlap when only the 237 genes considered as specific in our RLR screen are included.

The individually arrayed lentiviral-based short hairpin RNA (shRNA) human library (Mission TRC-Hs1.0 from Sigma-Aldrich) produced in-house was used to knockdown the expression of _∼_15,000 human genes by RNA interference (RNAi) combining 3 shRNAs per gene. HEK 293T cells stably expressing the firefly luciferase gene under the control of the *IFNB1* promoter was used to monitor type I IFN expression in response to SeV infection (Figure 1A). A robust 100-fold increase in luminescence was obtained upon infection and the cell assay was used to screen the shRNA human library by silencing one gene per well in 96-well plate format (Figure S1). The primary genome-wide screen was performed with control shRNAs on each plate targeting the positive regulator (PR) MAVS gene, which produced a tenfold decrease in luminescence, and the negative regulator (NR) NLR family member X1 (NLRX1) gene [11], which increased the luminescence by approximately twofold (Figure S1A). As control, the transduction of cells with non target sequence (NT) shRNA-expressing lentiviral particles was evaluated on SeV-induced *IFNB1* promoter activity three days post-transduction. Similar inductions in luciferase and mRNA levels were detected in control transduced cells compared to non-transduced cells demonstrating that infection of cells with shRNA-expressing lentivirus is not contributing to the SeV-mediated antiviral responses. Following statistical analyses of the data using the strictly standardized mean difference (SSMD) as a cutoff [12], we selected 292 potential PR whose silencing significantly decreased the *IFNB1* reporter activity (SSMD≤−1.314, approximately ≥2 SDs from the plate median) and 289 NR whose silencing significantly increased the *IFNB1* reporter activity (SSMD≥1.662, approximately ≥2.5 SDs from the plate median). For the 581 gene hits, we produced five independent shRNA-expressing lentiviruses that were tested individually in confirmation and secondary screens (Figure 1B). These secondary assays relied on the activation of the RLR pathway at different landmarks of the signaling cascade, through transfection of polyI∶C (dsRNA mimetic) to active RIG-I directly, overexpression of MAVS leading to its constitutive activation, and overexpression of IRF3(5D), a phosphomimetic mutant of IRF3 constitutively targeted to the nucleus, to activate transcription at the *IFNB1* promoter (Figure 1C). The inhibition profile of each shRNA in secondary screens allowed to classify gene hits within the RLR signaling cascade in four functional groups: I - SeV specific, II - cytoplasmic dsRNA sensing, III - MAVS-dependent signaling, IV - nuclear import or transcription factor-dependent process (Figure 1D for validation of secondary assays with control genes). Secondary screening resulted in the identification of 237 gene hits (132 PR and 105 NR) that modulate specifically the reporter activity at the *IFNB1* promoter without affecting reporter expression from a nonimmune-related endogenous elongation factor 1 alpha (EF1α, also known as EEF1A1) promoter (Figures 1B and 1C). Further prioritizing studies included the transcriptional modulation of virus-induced endogenous *IFNB1* mRNA synthesis with at least two shRNAs, which emphasized 114 gene hits, 80 PR and 34 NR (Figures 1E, 1F and S2). Finally, qRT-PCR validation showed a knockdown efficiency of greater than 40% (median knockdown efficiency of 70%) with two independent shRNAs for 59 gene prioritized gene hits (Figure 1F and see Table S1 for the complete set of primary, secondary and qRT-PCR data). The reliability of the screening approach was confirmed by detection of 10 out of the 114 gene hits (9.6%) previously associated to innate immunity, including the signaling components RIG-I (PR group II), MAVS (PR group III), IRF3 (PR group IV) and the NF-κB subunits REL and RELA (both PR group IV).

### Bioinformatics analysis of gene hits

To create a more comprehensive view of all prioritized gene hits in the regulation of antiviral innate immune responses, we performed a bioinformatics meta-analysis that integrates the data of our functional genomics screen with previous functional and proteomic studies. Gene ontology (GO) molecular functions of the 114 gene hits identified enrichment for 10 statistically significant terms encompassing expected terms: regulation of the NF-κB cascade, response to virus, RLR signaling pathway, regulation of cytokine production and antiviral defense, as well as the interesting functional groups: nuclear pore, proteasome and spliceosome (Figure 1G). Additional microarray analysis was performed to identify genes that were induced by more than twofold following SeV infection or IFN-α treatment. Based on our gene profiling analysis and previously described ISGs [13], 17 out of 237 gene hits were identified as ISGs (Figure 1H). Globally, identified gene hits were significantly enriched in ISGs (12 out of 114 genes or 10.5%) compared to a 2.0% representation of the human genome. The analysis also identified four nuclear pore complex (NPC) genes: nucleoporin 93 kDa (NUP93), RAN member RAS oncogene family (RAN), nucleoporin like 1 (NUPL1) and Exportin 1 (CRM1 homolog, yeast) (XPO1) within the group IV that significantly reduced virus-induced IFNB1 transcription upon individual gene perturbation. This example highlights a central role of XPO1-containing protein complex in innate antiviral response that is targeted by several viruses. Indeed, XPO1 is implicated in the nuclear export of the 60S ribosomal RNA subunit (rRNAs) [13] and specific mRNAs (IFNA1 mRNA [14]), and also contributes to the transport of viral proteins and RNA transcripts [15]. Interestingly, XPO1-dependent export of HIV-1 Rev protein required the DExD box RNA helicase DDX3 [16], which plays a role in type I IFN induction through TBK1/IKBKE (also known as IKKε)-mediated phosphorylation of IRF3 [17], [18].

In order to identify common proteins functioning as sensors of different danger signals and effectors of innate response, and as host antiviral restriction factors targeted by viruses, we compared gene hits of our RLR screen to those of biologically relevant RNAi screens for pathogen replication (HIV [19]–[21], HCV [22], [23], Influenza [24]–[26] and West Nile virus [27]) and for Nucleotide-binding and oligomerization domain (NOD)–like receptor (NLR) response [28] (Figure 1I). These screens were performed with different genome-wide RNAi libraries, cell lines, reporter genes and experimental designs. Nevertheless, the overlap analysis identified 58 putative modulators of virus-induced innate responses that are also associated to virus infection, and 9 common modulators of the RLR- and NLR-mediated recognition and signaling pathway (Supplementary Table 2). The overlapping genes are significantly enriched for the functional groups proteasome and spliceosome, and for the RLR, NLR and cancer pathways. Our comparison also highlights four shared genes between the three types of RNAi screen: RELA/p65, central component of the NF-κB pathway; RIPK2 a serine/threonine protein kinase containing a C-terminal caspase activation and recruitment domain (CARD) acting as a potent activator of NF-κB [29]; KLF6, a zinc finger protein recently identified as a critical transcription factor required for the innate immune response against influenza virus [30]; and CYP2U1, a hydroxylase member of the cytochrome P450 superfamily of enzymes involved in the synthesis of lipids with a potential physiological role in fatty acid signaling processes in brain and thymus [31]. Our genome-wide shRNA screen complements previously available immune and viral replication screens to enrich the global interaction networks of innate immune response, which could identify targets for the development of broad-spectrum anti-infective agents.

### Secretion of WNT2B and WNT9B acts in a feedback inhibition of SeV-induced *IFNB1* transcription

**Figure 2:**
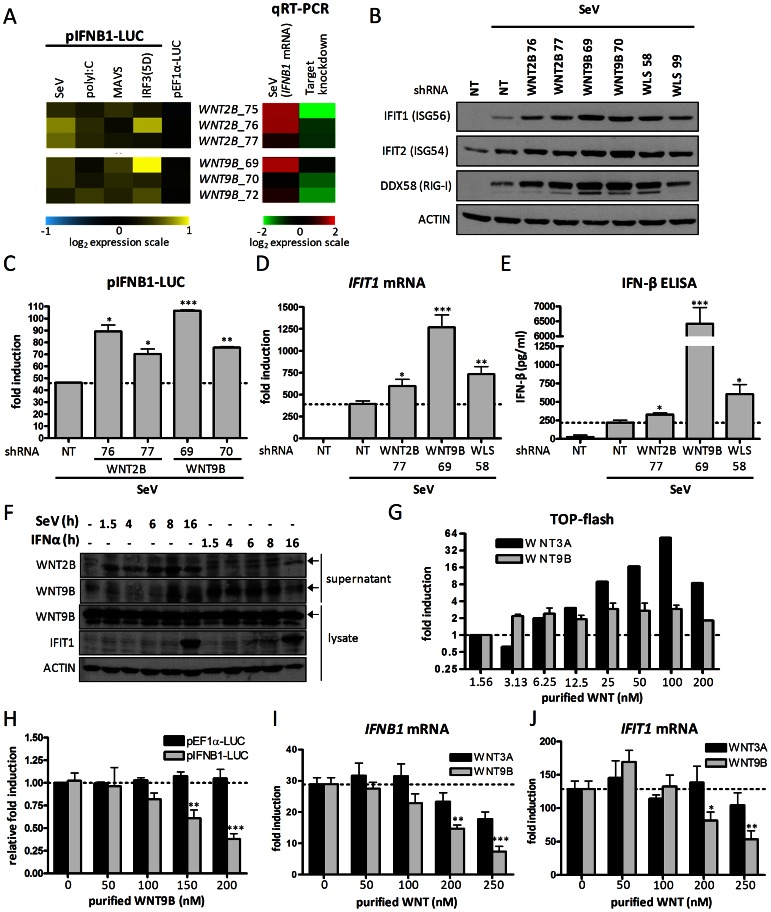
WNT2B and WNT9B ligands as novel negative regulators of antiviral innate immunity. (A) Heat map indicating log_2_ effect of silencing WNT2B and WNT9B genes with 3 independent lentivirus-expressing shRNAs (left) and qRT-PCR validation of endogenous *IFNB1* mRNA levels and of target gene knockdown efficiency (right). (B) Immunoblot analysis of IFIT1, IFIT2 and DDX58 in WNT2B, WNT9B and WLS knockdown HEK 293T cells (two-independent shRNAs per gene) following infection with SeV for 16 hours. (C) *IFNB1* promoter-driven luciferase activity in HEK 293T cells treated as described in (B). (D) Fold induction of *IFIT1* mRNA levels in HEK 293T cells treated as described in (B). qRT-PCR determination represents the average mRNA RQ normalized versus ACTIN and HPRT1 mRNA. (E) ELISA quantification of secreted IFN-β protein in supernatant of HEK 293T cells treated as described in (B). (F) Immunoblot analysis of HEK 293T supernatants and lysates at various time points in cells overexpressing WNT2B and WNT9B and infected with SeV or treated with IFN-α. The specific bands are indicated with arrows. (G) Fold induction of CTNNB1-TCF/LEF dependent reporter activity (TOP-flash) with addition of purified WNT3A or WNT9B proteins to HEK 293T cell supernatants for 16 hours. (H) Relative fold induction of *IFNB1*- or *EF1α*-driven reporter activity with dose-dependent addition of purified WNT9B protein to cell supernatants of SeV infected HEK 293T cells for 16 hours. (I–J) Fold induction of *IFNB1* (I) or *IFIT1* (J) mRNA levels following dose-dependent addition of purified WNT3A or WNT9B proteins to cell supernatants of SeV infected HEK 293T cells for 16 hours. qRT-PCR determination represents the average mRNA RQ normalized versus ACTIN and HPRT1 mRNA. P values<0.05 (*), <0.01 (**) or <0.001 (***) are indicated.

The RNAi screen identified gene hits encoding WNT ligands that modulate virus-induced innate response. WNT ligands are a family of highly conserved secreted glycoproteins that regulate multiple processes in development and tissue homeostasis. These processes are mediated through canonical WNT/β-catenin (CTNNB1) and non-canonical WNT pathways [32], [33]. In recent years, few studies have reported a role of WNT pathway in the regulation of inflammation. In *drosophila*, WntD was shown to act as a feedback inhibitor of the NF-κB homolog Dorsal in response to bacterial infection [34]. More recently, Wnt2 and Wnt11 were shown to inhibit bacterial-induced inflammation in intestinal epithelial cells in mouse [35], [36]. We identified two highly prioritized genes encoding WNT2B (also called WNT13, NR group IV) and WNT9B (also called WNT15 and WNT14B, NR group IV), which upon gene silencing significantly increased gene transcription of the pIFNB1-LUC reporter construct and of the endogenous *IFNB1* promoter in SeV-infected cells (Figure 2A). The knockdown of both WNT ligands increased pIFNB1-LUC expression induced by transfected polyI∶C and overexpression of MAVS and of IRF3(5D), consistent with a regulatory role of the antiviral response in a feedback inhibition loop (Figure 2A). SeV infection led to a significant increase of the virus-induced representative proteins IFIT1 (ISG56), IFIT2 (ISG54) and DDX58 (RIG-I) at 16 hours post-infection with knockdown of WNT2B or WNT9B by two independent shRNAs (Figure 2B and Figure S3 for knockdown efficiency). The increased pIFNB1-LUC expression upon WNT2B or WNT9B silencing is further illustrated by enhancement of *IFIT1* mRNA induction levels, and more importantly by increased levels of IFN-β protein in the supernatant of infected cells (Figures 2C–E). As an example, we observed in WNT9B knockdown cells versus control cells transduced with shRNA NT more than 6,000 pg/mL of secreted IFN-β protein (versus 250 pg/mL), a 650-fold in *IFNB1* mRNA (versus 100-fold), a 800-fold in IFIT1 mRNA (versus 250-fold), and a 100-fold increase in NF-κB-driven *TNF* mRNA levels (versus 50-fold in control shRNA NT cells) (Figures 2E and S4). Co-transfection of shRNA-resistant expression vectors for WNT2B or WNT9B rescued the knockdown by decreased pIFNB1-LUC expression excluding shRNA off-target effects (Figure S5).

We then investigated the modulation in gene expression of the two WNT ligands following virus infection. Since WNT2B and WNT9B mRNA levels are barely inducible (less than 2-fold) in all conditions tested (Figure S6), we evaluated the secretion of ectopically expressed WNT2B and WNT9B upon SeV infection and IFN-α treatment (Figure 2F). Our data revealed a secretion of WNT2B that build-up until 8 hours post-infection, while no secretion was observed with IFN-α treatment. The virus-induced secretion of WNT2B was also confirmed in cervical-derived HeLa and hepatoma Huh7 human cells (Figure S7A). In contrast, WNT9B is rapidly secreted with IFN-α treatment, while its secretion is merely initiated 8 hours post SeV infection, a time point correlating with virus-induced *IFNB1* expression (Figures 2F and S6). We then confirmed the requirement of virus-induced WNT secretion for the negative regulation of *IFNB1* transcription by perturbation of wntless homolog (Drosophila) (*WLS*) gene encoding a conserved membrane protein dedicated to WNT secretion in signaling cells [37]–[39]. Indeed, we observed that silencing of WLS reduced WNT secretion (Figure S7B), and increased the expression of IFIT1, IFIT2 and DDX58 proteins, *IFIT1* mRNA levels and secretion of IFN-β protein induced by SeV in HEK 293T cells (Figures 2B, 2D, 2E and S3 for knockdown efficiency ).

Finally, to confirm a direct role of WNT ligand in the negative regulation of *IFNB1* mRNA expression, we incubated SeV-infected cells with purified WNT proteins. As expected, purified WNT9B decreased virus-induced pIFNB1-LUC activity, as well as mRNA levels of *IFNB1* and of *IFIT1* from endogenous promoters, by up to threefold in dose-response without affecting the constitutive EF1α promoter (Figures 2H–J). In control experiments, purified WNT9B weakly induced the CTNNB1-dependent T-cell factor/lymphoid enhancer factor (TCF/LEF)-promoter driven reporter activity (M50 Super 8×TOP-flash [40]) by less than 3-fold while purified WNT3A protein achieved a 60-fold induction of reporter activity (Figure 2G). In addition, purified WNT3A weakly decreased SeV-induced *IFNB1* and *IFIT1* transcription and at concentrations (250 nM) well above those required to promote CTNNB1 TCF/LEF-dependent reporter activity (compare Figures 2G with Figures 2I and 2J). Overall, these results strongly support a specific effector role of virus-induced secretion of WNT2B and WNT9B ligands in innate immunity, acting in a feedback inhibition of IRF3- and NF-κB-dependent *IFNB1* response to virus infection.

### CTNNB1 negatively regulates *IFNB1* gene expression

**Figure 3:**
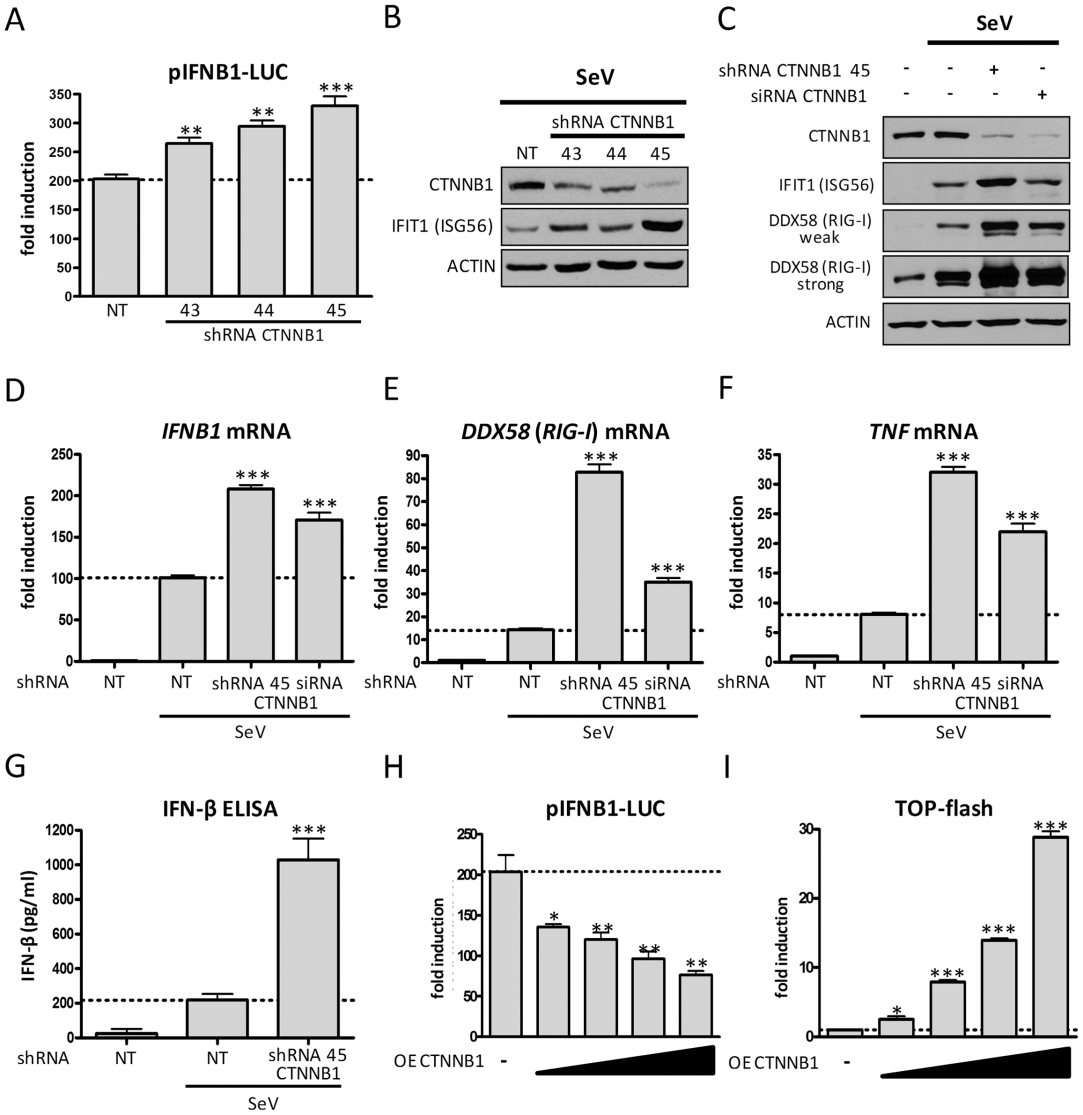
CTNNB1 is a negative regulator of antiviral innate immunity. (A) *IFNB1* promoter-driven luciferase activity in HEK 293T cells transduced with lentivirus-expressing shRNA NT (control) or 3 shRNAs targeting CTNNB1 for four days and subjected to SeV infection for 16 hours. (B) Immunoblot analysis of CTNNB1 and IFIT1 in HEK 293T cells transduced with 3 lentivirus-expressing shRNAs targeting CTNNB1 and infected with SeV. (C) Immunoblot analysis of CTNNB1, IFIT1 and DDX58 in SeV-infected HEK 293T cells previously transduced with a shRNA or transfected with a pool of four siRNAs targeting CTNNB1 for three days. (D–F) Fold induction of *IFNB1* (D), *DDX58* (E) and *TNF* (F) mRNA levels in HEK 293T cells treated as described in (C). qRT-PCR determination represents the average mRNA RQ normalized versus ACTIN and HPRT1 mRNA. (G) ELISA quantification of secreted IFN-β protein in supernatant of HEK 293T cells treated as described in (A). (H–I) Fold induction of *IFNB1* promoter-driven luciferase activity (H) and of CTNNB1-TCF/LEF dependent reporter activity (TOP-flash, I) following dose-dependent transfection of CTNNB1-expressing plasmid (50, 100, 150 and 200 ng) for 48 hours in SeV-infected HEK 293T cells.

In a canonical WNT pathway, WNT ligands bind to cell-surface receptors of the frizzled family receptor (FZD) and low density lipoprotein receptor-related protein (LRP) 5/6, activate dishevelled (DVL) and ultimately result in stabilization of cytoplasmic CTNNB1 and its nuclear translocation. The process is mediated by the WNT/LRP-signalosome through recruitment at cell membrane of glycogen synthase kinase 3β (GSK3B) and AXIN, disrupting the cytoplasmic GSK3B/AXIN/adenomatous polyposis coli (APC) destruction complex of CTNNB1. WNT-mediated activation of CTNNB1 promotes specific gene expression via interaction with TCF/LEF transcription factors. While SeV infection per see do not induce TCF/LEF transcription (data not shown), we investigated the contribution of CTNNB1 on SeV-mediated IFNB1 and IFIT1 induction to better understand mechanistically the role of WNT2B/WNT9B in regulation of innate immunity. The knockdown of *CTNNB1* gene produced the expected enhancement in virus-induced pIFNB1-LUC reporter activity and in IFIT1 protein levels (Figures 3A and 3B). Knockdown of *CTNNB1* gene by specific shRNA and siRNAs also enhanced induction of DDX58 protein levels, of mRNA levels of *IFNB1*, *DDX58* and *TNF* from endogenous promoters and notably increased secretion of IFN-β protein (Figures 3C–G and Figure S3 for knockdown efficiency). Conversely, the ectopic expression of CTNNB1 resulted in diminution of virus-induced *IFNB1* transcription in a dose response manner, while promoting the TCF/LEF-dependent reporter activity in control experiments (Figures 3H and 3I). To test the potential additive effect of WNT2B and WNT9B knockdown and to confirm that these WNTs act through CTNNB1 in regulating innate antiviral immunity, we performed double knockdown experiments. We showed an additive effect of the knockdown of WNT2B and WNT9B together in enhancing IFNB1 induction following SeV infection (Figure S8). However, the knockdown of WNT2B or WNT9B in combination with CTNNB1, as expected, do not further increase IFNB1 induction when compared to the knockdown of CTNNB1 alone (Figure S8).

**Figure 4:**
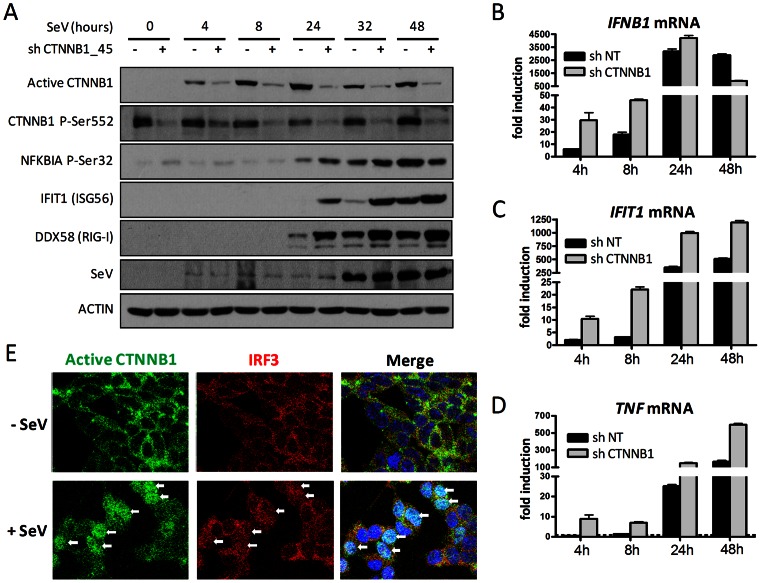
SeV infection induces CTNNB1 stabilization and nuclear translocation. (A) Immunoblot analysis of dephosphorylated active CTNNB1 at Ser37/Thr41, CTNNB1 phosphorylated at Ser552, NFKBIA (IκBα) phosphorylated at Ser32, IFIT1, DDX58 and SeV protein HN of HEK 293T transduced with lentivirus-expressing shRNA 45 targeting CTNNB1 and shRNA NT (control) for four days and subjected to SeV infection for 0, 4, 8, 24, 32 or 48 hours. (B–D) Fold induction of *IFNB1* (B), *IFIT1* (C) and *TNF* (D) mRNA levels in HEK 293T cells treated as described in (A). qRT-PCR determination represents the average mRNA RQ normalized versus ACTIN and HPRT1 mRNA. (E) Confocal analysis of HEK 293T cells using Hoechst, anti-CTNNB1 active form and anti-IRF3 antibodies without virus infection or following 16 hours infection with SeV. Nuclear detection of IRF3 and CTNNB1 is identified by white arrows in infected cells.

We then confirmed the stabilization of endogenous CTNNB1 upon SeV infection in a kinetics study. Indeed, immunoblot analysis of infected cell extracts demonstrated the progressive increase in activated CTNNB1 (dephosphorylated on Ser37 or Thr41) from 4 to 24 hours post-infection, followed by a slight decrease at 48 hours (Figure 4A). The stabilization of CTNNB1 following SeV infection is also observed following ectopic expression of a FLAG-tag CTNNB1 protein (see Figure S12B). Importantly, the kinetic showed no induction in the basal level of phosphorylated CTNNB1 Ser552 upon SeV infection. This is in contrast to the LRRFIP1-dependent phosphorylation of CTNNB1 at Ser552 upon infection with Listeria monocytogenes, which promotes its transcriptional activation required for IFNB1 production in mouse macrophages [41]. Moreover, the kinetics study of SeV infection performed in CTNNB1 knockdown cells clearly showed that blocking CTNNB1 accumulation upon gene silencing correlated with increased activation of the antiviral response as demonstrated by the protein levels of IFIT1 and DDX58, as well as phosphorylated levels of NFKBIA (IκBα) at serine 32, an indicator of NF-κB activation (Figure 4A). IFIT1 and DDX58 proteins increased from 24 to 48 hours post-infection while phosphorylated NFKBIA increased up to 32 hours post-infection in CTNNB1 knockdown cells. As a control, we demonstrated that this increase in innate antiviral response is not a consequence of increase viral replication, since the levels of SeV hemagglutinin-neuraminidase (HN) viral proteins are unchanged in CTNNB1 knockdown cells.

In addition, qRT-PCR quantification of *IFNB1*, *IFIT1* and *TNF* mRNA levels showed increase in their promoter activity up to seven-fold in CTNNB1 knockdown cells (Figures 4B, 4C and 4D). Interestingly, a more rapid decline of the *IFNB1* mRNA level is observed at 48 hours post-infection (Figure 4B) compared to *IFIT1* and *TNF* mRNA levels, which remained higher at this time point (Figure 4C and 4D). In immunofluorescence experiments, we specifically followed the cytoplasmic CTNNB1 regulated by the GSK3B destruction complex using the antibody specific to the activated/dephosphorylated CTNNB1. Following infection, CTNNB1 is detected in the nucleus of SeV-infected HEK 293T cells, similarly to IRF3 (Figure 4E). Similar results were observed in infected A549 cells together with a nuclear co-staining of CTNNB1 and NF-κB (Figure S9). Altogether, our data strongly suggest that the stabilization and/or accumulation of CTNNB1 in the nucleus upon virus-induced WNT secretion triggered a CTNNB1-dependent negative feedback loop to reduce antiviral innate immune responses.

### GSK3 inhibitors block innate response through CTNNB1 stabilization

**Figure 5:**
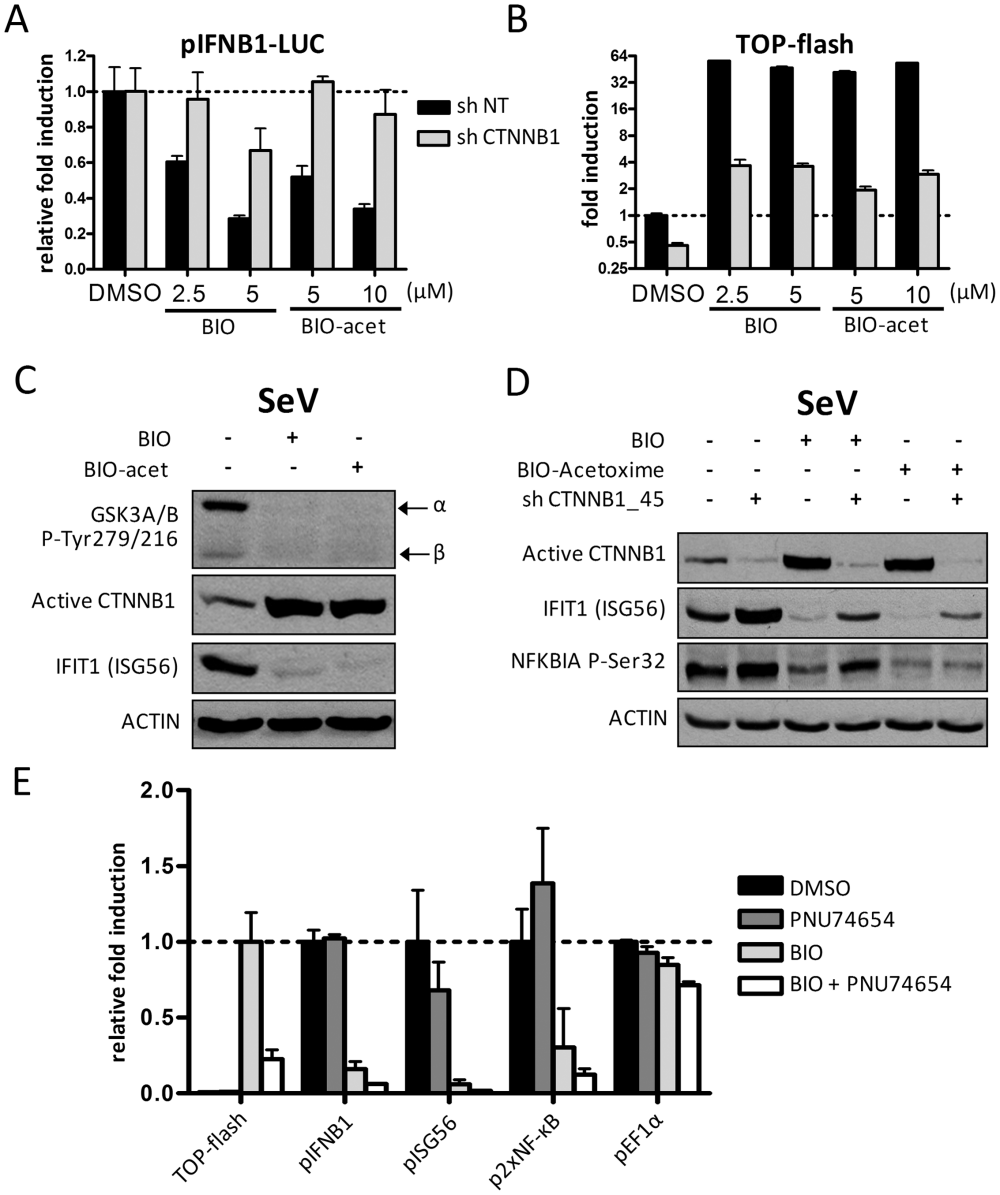
Pharmacological inhibition of GSK3 stabilizes an active CTNNB1 pool and negatively regulates antiviral innate immunity. (A–B) Relative fold induction of *IFNB1*-driven reporter activity (A) and fold induction of CTNNB1-TCF/LEF dependent reporter activity TOP-flash (B) in HEK 293T cells transduced with lentivirus-expressing shRNA NT (control) or shRNA 45 targeting *CTNNB1* for four days and subjected to GSK3 inhibition with BIO (5 µM) or BIO-acetoxime (10 µM) and SeV infection for 16 hours. (C) Immunoblot analysis of autophosphorylated GSK3α/β at Tyr279/216, dephosphorylated active CTNNB1 at Ser37/Thr41 and IFIT1 in HEK 293T cells treated with GSK3 inhibitors BIO (5 µM) or BIO-acetoxime (10 µM) and infected with SeV for 16 hours. (D) Immunoblot analysis of dephosphorylated active CTNNB1 at Ser37/Thr41, IFIT1 and phosphorylated NFKBIA (IκBα) at Ser32 in SeV-infected HEK 293T cells treated as described in (A–B) and treated with BIO (5 µM) or BIO-acetoxime (10 µM). (E) Relative fold induction of TOP-flash, *IFNB1*, *ISG56*, *NF-κB* and *EF1α*-driven reporter activity in HEK 293T cells subjected to GSK3 inhibition with BIO (5 µM), TCF/LEF interaction inhibition with PNU74654 (6 µM) or both inhibitors prior to SeV infection for 16 hours.

Since the inhibition of GSK3B catalytic activity results in CTNNB1 cytoplasmic accumulation, we then investigated a role of GSK3 in the negative regulation of *IFNB1* induction (Figure 5). The inhibition of GSK3A/B activity with specific inhibitors BIO (5 µM) and BIO-acetoxime (10 µM) was confirmed by lack of autophosphorylation at Tyr279/Tyr216, respectively, using phospho-specific antibodies (Figure 5C). As expected, pharmacological inhibition of GSK3 increased active CTNNB1 levels (dephosphorylated on Ser37 or Thr41) that correlated with the CTNNB1 TCF/LEF-dependent reporter activity (Figures 5B and 5C). More importantly, the inhibition of GSK3 resulted in a drastic reduction of virus-induced *IFNB1* transcription as well as expression of IFIT1 and of NFKBIA phosphorylation at serine 32 when compared to untreated SeV-infected cells (Figures 5A, 5C and 5D). As GSK3 phosphorylates multiple substrates other than CTNNB1 affecting different pathways, we further demonstrated that the decrease in virus-dependent induction of pIFNB1-LUC, IFIT1 and phosphorylated NFKBIA by GSK3 inhibitors is specifically abolished in CTNNB1 knockdown cells (Figures 5A and 5D). Parallel transcriptional changes were observed with *IFNB1*, *IFIT1* and *TNF* mRNA levels (Figure S10A–C). Finally, similar restoration of the antiviral response was observed in GSK3 inhibitor-treated CTNNB1 knockdown A549 cells following SeV infection (Figure S10D). In control experiments, increased CTNNB1 TCF/LEF-dependent reporter activity with pharmacological inhibition of GSK3 was also specifically abrogated by knockdown of CTNNB1 (Figure 5B). To verify if the inhibition of the innate antiviral response observed upon pharmacological stabilization of CTNNB1 was a consequence of its transcriptional activity through the induction of TCF/LEF-dependent genes, we used the CTNNB1-TCF interaction inhibitor PNU74654 [42]. The addition of PNU74654 (6 µM) reduced the GSK3 inhibitor-mediated TOP-flash induction by 70%, without restoring innate antiviral response to SeV infection as determined by pIFNB1-LUC, pISG56-LUC and p2xNF-κB-LUC reporter activities (Figure 5E). This experiment rule out a role of TCF/LEF inducible genes by the canonical WNT3A pathway (involved in embryonic development or cell fates during normal development) as part of the negative regulation of innate antiviral immunity. Altogether, the results strongly suggest a key role of GSK3 catalytic activity in a feedback inhibition of the antiviral innate immune response mediated by a canonical-like WNT2B/WNT9B/CTNNB1 signaling pathway.

### WNT/CTNNB1 pathway negatively regulates innate immune response in primary human cells

**Figure 6:**
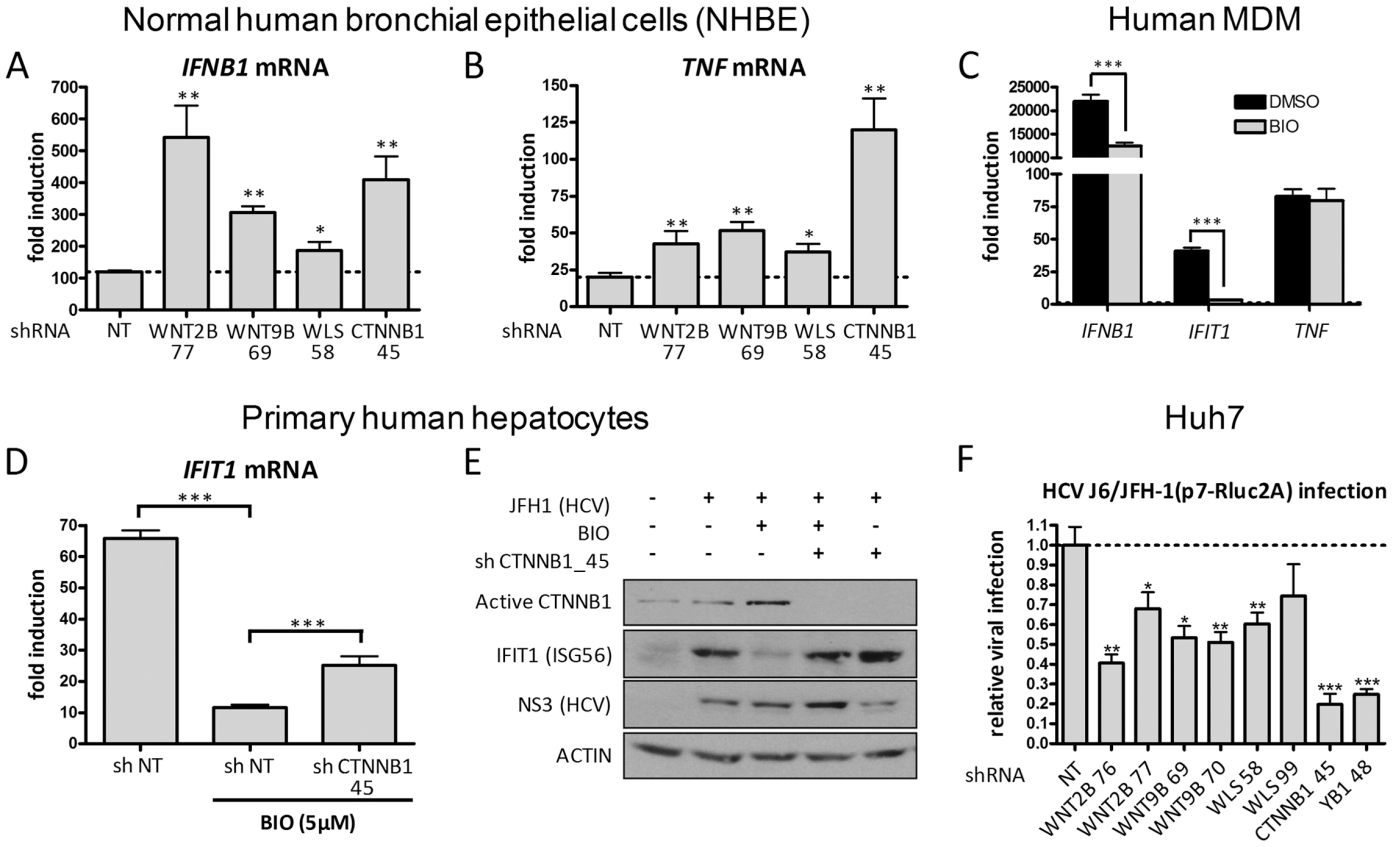
WNT/CTNNB1 signaling acts as a negative regulator of the innate immune response in primary human cells. (A–B) Fold induction of *IFNB1* (A) and *TNF* (B) mRNA levels in NHBE cells transduced with lentivirus-expressing shRNA 77 targeting *WNT2B*, shRNA 69 targeting *WNT9B*, shRNA 58 targeting *WLS*, shRNA 45 targeting *CTNNB1* and NT shRNA in control cells for four days and infected with SeV for 16 hours. (C) Fold induction of *IFNB1*, *IFIT1* and *TNF* mRNA levels in human monocyte-derived macrophages subjected to GSK3 inhibition with BIO (5 µM) and SeV infection for 4 hours. qRT-PCR determination represents the average mRNA RQ normalized versus ACTIN and HPRT1 mRNA. (D) Fold induction of *IFIT1* mRNA in primary human hepatocytes transduced with lentivirus-expressing shRNA NT or shRNA 45 targeting *CTNNB1* for four days and subjected to GSK3 inhibition with BIO (5 µM) and SeV infection for 5 hours. qRT-PCR determination represents the average mRNA RQ normalized versus ACTIN and HPRT1 mRNA. (E) Immunoblot analysis of dephosphorylated active CTNNB1 at Ser37/Thr41, IFIT1 and HCV NS3 protease in primary human hepatocytes either transduced with lentivirus-expressing shRNA 45 targeting CTNNB1 for four days and/or subjected to GSK3 inhibition with BIO (5 µM) and infected with Hepatitis C virus (HCV) strain JFH1 for 48 hours. (F) Huh7 cells transduced with shRNAs NT, WNT2B 76, WNT2B 77, WNT9B 69, WNT9B 70, WLS 58, WLS 99, CTNNB1 45 or YB1 48 (positive control) were infected with HCV J6/JFH-1(p7-Rluc2A) reporter virus. Following 96 hours of infection, Rluc was measure to assess HCV viral replication and results are represented as relative viral infection with shRNA NT treated cells arbitrarily set to 1. P values<0.05 (*), <0.01 (**) or <0.001 (***) are indicated.

The regulation of antiviral innate immunity by a WNT/CTNNB1 pathway was further investigated in primary cultures of normal human bronchial epithelial cells (NHBE), human monocyte-derived macrophages (MDM) and human hepatocytes. The negative regulation of SeV-induced innate response was demonstrated in NHBE cells as shown by the knockdown of WNT2B, WNT9B and CTNNB1, which increased *IFNB1* mRNA levels by up to five folds (Figure 6A) and *TNF* mRNA levels by up to six folds (Figure 6B). Significant increased *IFNB1* and *TNF* mRNA levels are also observed in WLS knockdown cells (1.5 to 2 folds). We demonstrated the key contribution of CTNNB1 in immune cells and showed that the treatment of MDM with GSK3 inhibitors led to a robust inhibition in *IFNB1* and *IFIT1* mRNA levels up to 90%, in accordance with results obtained in non immune cells (Figure 6C). However, *TNF* mRNA induction was systematically weakly or unaffected by GSK3 inhibitor treatment, demonstrating some differences in regulation of MDM compared to epithelial cells at 4 hours post-infection. We also confirmed in primary cultures of normal human hepatocytes that the pharmacological inhibition of GSK3 resulted in a drastic inhibition of virus-induced *IFIT1* transcription that is abrogated in CTNNB1 knockdown cells (Figure 6D). More importantly, we emphasized the contribution of the GSK3/CTNNB1 in regulation of innate immune response following hepatitis C virus (HCV) infection. Indeed, incubation of primary human hepatocytes with the full-length HCV strain JFH1 for 48 hours led to the induction of IFIT1 protein levels (Figure 6E). Using this model, we showed a drastic inhibition of IFIT1 levels when treating hepatocytes with the GSK3 inhibitor BIO, and in accordance with previous data, a restoration of IFIT1 levels in GSK3 inhibitor-treated CTNNB1 knockdown cells (Figure 6E). It is noteworthy that treatment with GSK3 inhibitor did not affect levels of HCV NS3 protease in these primary hepatocytes, confirming that the observed effect on IFIT1 protein is not related to an alteration of HCV infection at early time points. Upon knockdown of CTNNB1 however, the observed increase in IFIT1 protein levels and decrease in NS3 protein of HCV-infected primary hepatocytes prompted us to evaluate the quantitative effect of WNT/CTNNB1 signaling on HCV infection. Indeed, we observed a reduced HCV replication upon knockdown of WNT2B, WNT9B, WLS and CTNNB1 in Huh7 cells infected with the reporter HCV J6/JFH-1(p7-Rluc2A) virus (Figure 6F). Up to 80% inhibition of HCV infection was observed in CTNNB1 knocked down cells, a similar effect than the silencing of Y-box-binding protein-1 (YB1) in a positive control that we previously identified as a cofactor of HCV replication and assembly [43]. Altogether, these results confirm the role of a canonical-like WNT/GSK3/CTNNB1 pathway in a negative regulation of innate immunity upon viral infections of primary human cells including immune cells.

### Virus-induced CTNNB1 association with IRF3

**Figure 7:**
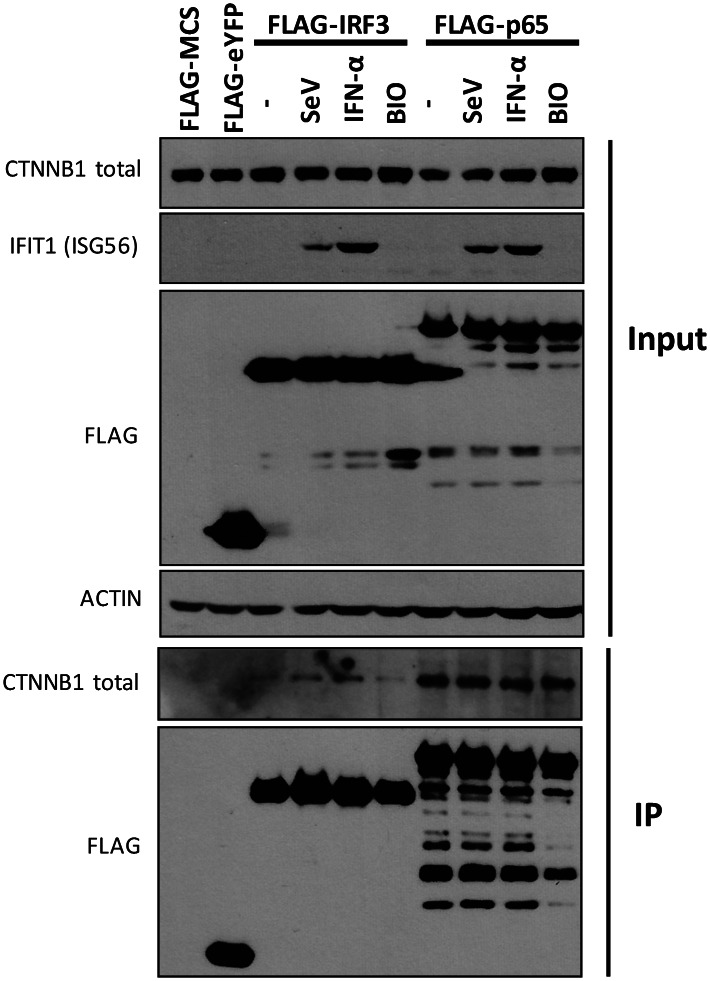
CTNNB1 associates with IRF3 and NF-κB subunit p65. HEK 293T cells were transfected with FLAG-MCS (control), FLAG-eYFP (control), FLAG-IRF3 or FLAG-p65 expressing plasmids for forty-eight hours. Cell extracts were prepared following 16 hours of treatment with IFN-α, GSK3 inhibitor BIO (5 µM) or SeV infection before being subjected to immunoprecipitation directed against FLAG. Cell extracts and immune complexes were analyzed by Western blotting using anti-FLAG and anti-CTNNB1 antibodies.

To elucidate the underlying mechanism by which activation of a canonical-like WNT/CTNNB1 pathway decreases innate antiviral responses by negatively regulating IRF3- and NF-κB- dependent production of IFN-β production, we determined if CTNNB1 physically interacts with IRF3 and NF-κB subunits by co-immunoprecipitation experiments. Immunoprecipitation followed by immunoblot analysis showed that SeV infection or treatment of cells with IFN-α increase the interaction of FLAG-IRF3 with endogenous CTNNB1 (Figure 7). In contrast, the observed interaction of NF-κB RELA/p65 subunit with endogenous CTNNB1 remains unchanged following these stimulations. Ectopic expression of FLAG-tagged IRF3, constitutively active IRF3(5D) and RELA/p65 further demonstrated an interaction with exogenous CTNNB1 in SeV-infected cells (Figure S12A). Reciprocal immunoprecipitation of FLAG-CTNNB1 in SeV-infected cells failed to detect interaction with endogenous IRF3 while confirmed the stable interaction with endogenous p65 (). Importantly, the sole increase in endogenous CTNNB1 by stabilization with a GSK3 inhibitor does not promote the interaction with IRF3 in the absence of viral infection (Figure 7 lane BIO). Altogether, these results suggest a direct role of a virus-induced interaction of CTNNB1 with IRF3 in the negative regulation of innate immunity.

## Discussion

In this study, SeV infection of human cells was used to induce a robust *IFNB1* expression and to mount an effective type I IFN innate immune response with induction of antiviral ISGs. This provides a model to study virus sensing, signal propagation and feedback regulation mechanisms at primary sites of virus infection. Here, a genome-wide gene silencing approach is used to uncover novel regulators of the antiviral innate immune response. The validation of the data will offer novel therapeutic avenues to control virus infection in pan-therapy, stimulate innate immunity in live virus vaccine, modulate oncolytic virus innate response in virotherapy and prevent excessive innate response in virus-mediated immune disorders. The ultimate objective of our work is to better define the human innate immunity signalosome and to unravel mechanisms associated with viral infection and virulence.

**Figure 8:**
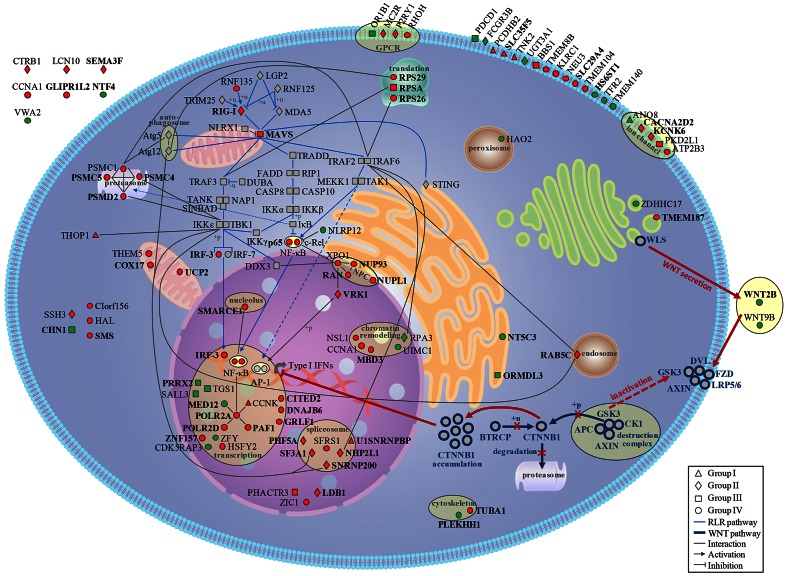
Cellular map of prioritized gene hits as potential modulators of innate immunity. Cellular mapping of gene products of 96 gene hits out of 114 that modulated endogenous *IFNB1* expression either as Positive Regulators (71 PRs: Red) or as Negative Regulators (25 NRs: green) of the signaling pathway following SeV infection. PRs and NRs were positioned on a cell map and classified according to sub-cellular compartments and cellular function using annotation information from Gene Ontology, KEGG pathway, Uniprot, NCBI and Ingenuity pathway Analysis (IPA). The four functional groups are represented by different symbols: I - SeV specific (triangle), II - cytoplasmic dsRNA sensing (diamond), III - MAVS-dependent signaling (square) and IV - Nuclear import or transcription factor-dependent process (circle). Gene hits with knockdown efficiency confirmed by qRT-PCR with at least two independent shRNAs are highlighted in bold (41 PRs and 9NRs). The RLR and WNT signaling pathways are represented in light and dark blue respectively. Virus-induced activation of the WNT/CTNNB1 canonical-like pathway in feedback inhibition of antiviral innate responses is indicated in red.

We now report the data of the first RNAi screen assessing the silencing effect of _∼_15,000 human genes on transcriptional activity of the *IFNB1* promoter during SeV infection. We identified 237 gene hits that significantly modulated antiviral innate response in stringent confirmation and validation processes (Table S1). By designing secondary assays, gene hits were further classified according to a negative or positive action of encoded gene products within the *IFNB1* signaling pathway from virus recognition to IFNB1 expression. From the epistasis analysis, data with endogenous *IFNB1* promoter and gene silencing efficiency analysis, we identified 114 highly prioritized genes. The subcellular localization of proteins encoded by 96 of these prioritized gene hits is depicted in Figure 8 and includes the network connectivity using annotation information of various bioinformatics resources. Our study provides a comprehensive view of the regulation of innate immune responses by the RLR pathway, and complements the published RNAi screen by Yeretssian *et al.* who identified potential regulators of the nucleotide-binding and oligomerization domain (NOD) pathway, a cytosolic PRR sensing peptidoglycans [28].

In this study, we provide evidence for novel functional links in the regulation of IFNB1 and ISGs expression by components of a WNT pathway in virus-infected cells. We identified WNT2B and WNT9B as negative regulators of *IFNB1* transcription and IFNB1 protein production acting downstream of IRF3(5D) activation of the innate immune response. We found that secretion of WNT proteins is induced by virus infection or IFN-α treatment and confirmed the requirement of WLS secretory function in a WNT-mediated regulatory role. We demonstrated that WNT secretion most likely results from virus-induced release of pre-existing intracellular pools rather than increased gene transcription. We also provided direct evidences that incubation of infected cells with purified WNT9B, but not WNT3A, reduced SeV-mediated *IFNB1* and *IFIT1* mRNA induction without significantly altering TCF/LEF-mediated gene transcription. This is further supported by the fact that SeV infection by itself does not induce transcription of TCF/LEF genes as measured by the TOP-flash reporter assay, and that a CTNNB1-TCF interaction inhibitor (PNU74654) has ruled out a role of TCF/LEF inducible genes in the regulation of innate immunity. These results prompt us to perform a biased RNAi screen targeting components of the WNT pathway, which included WNT ligands, FZD receptors and LRP co-receptors. WNT2B and WNT9B were confirmed as the only ligands acting significantly on virus-mediated *IFNB1* transcription among 16 tested WNT ligands (data not shown). We also observed a negative regulation phenotype for LRP5/6 and FZD5 knockdown cells (described as Wnt2b receptor [44]), which is coherent with activation of a WNT signalosome by WNT2B/WNT9B binding to trigger downstream signaling events in SeV-infected cells.

In order to uncover mechanistically the signaling pathways associated with WNT-receptor interaction and feedback inhibition of antiviral innate immune response, we investigated a canonical-like WNT pathway that culminates in CTNNB1 accumulation. Coherently, we found that viral infection induces the accumulation of an active form of CTNNB1 and that *CTNNB1* gene silencing increases SeV-induced *IFNB1*, *IFIT1* and *TNF* expression, as well as NFKBIA (IκBα) phosphorylation at serine 32 (P-Ser32) to release NF-κB inhibition. Conversely, GSK3 inhibition and CTNNB1 overexpression inhibited their induction. We also confirmed key observations in primary human cells, including NHBE epithelial cells, hepatocytes and MDM immune cells. A diminished IRF3-dependent and NF-κB-dependent antiviral responses upon gene silencing or inhibitor-treated cells were observed in all cell types with the exception of MDM for which *TNF* gene is unaffected by GSK3 inhibitor treatment at early time points. This is unexpected as GSK3 is generally required for pro-inflammatory cytokine production in immune cells [45] and may reflect a differential regulation of the IRF3- and NF-κB-dependent responses by a WNT pathway following RLR stimulation in MDM. Finally, in addition to SeV infection, the contribution of GSK3/CTNNB1 in feedback inhibition of innate immunity was further extended to HCV infection of primary human hepatocytes, suggesting a regulatory role in various types of viral infections. The activation of CTNNB1 in primary hepatocytes infected with HCV (Figure 6E) was previously reported [46]. However, this was mediated by the release of CTNNB1 from the E-cadherin complex and not from activation of a WNT signaling pathway. In this study, activation of the AKT/CTNNB1 pathway with phosphorylation of CTNNB1 at Ser552 induces epithelia-mesenchymal-transition in HCV-infected hepatocytes [46]. Whether this could be exploited by HCV to decrease antiviral innate immune response and allow increased viral replication remains to be demonstrated. Nevertheless, this is strongly supported by the knockdown of CTNNB1 that increases IFIT1 synthesis in HCV infected primary hepatocytes, while diminishing viral replication (Figure 6E–F).

Previous studies have reported anti-inflammatory properties of GSK3 inhibitors [47]–[53] but few have suggested CTNNB1 as the effector protein [54]–[57]. In addition, CTNNB1 has been involved in a GSK3-independent pathway through protein kinase C (PKC), AKT1 or LRRFIP1-dependent activation to positively or negatively regulate innate immunity [41], [58]–[60]. Yang *et al.* showed that CTNNB1 positively regulates *Listeria monocytogenes*-induced IFNB1 production in mouse macrophages via a MAVS-independent pathway, acting as a co-activator of IRF3 that promote recruitment of p300 to the *IFNB1* enhanceosome. They found that LRRFIP1 interacts with CTNNB1 to promote phosphorylation of CTNNB1 at Ser552. In our study, the role of CTNNB1 as a feedback inhibitor (co-repressor) may be explained by differential post-translational modifications of CTNNB1, which upon activation of a canonical-like WNT pathway promote a negative regulation of the innate response by antagonizing IRF3 function. Our data showed that SeV-induced interaction of IRF3 with CTNNB1 does not correlate with phosphorylation of CTNNB1 at Ser552. Whether this interaction is phosphorylation-dependent is unknown at this time but may also involve regulation by other post-translational modifications such as PKC-dependent deacetylation of CTNNB1 at Lys49 by HDAC6 for its nuclear translocation and promoter binding [59]. Nevertheless, the activation of a canonical-like WNT/CTNNB1 pathway by virus infection must play its regulatory role in the late stage of IRF3 transcriptional activity in the infected cells via different scenario for a CTNNB1-IRF3 protein complex: import inhibition into the nucleus, increased nuclear export, increased protein degradation or as repressor of transcription. The effect of CTNNB1 as a negative regulator of *IFNB1* could also be explained by the observed negative regulation of the NF-κB signaling pathway (Figures 3–6, S10 and S11) and as previously reported [56]. Interestingly, the recent observation of the involvement of DDX3 in the activation of the WNT/CTNNB1 pathway [61] may also contribute to a negative regulation of *IFNB1* by its recruitment to the plasma membrane and inhibition of its role in the RLR pathway through TBK1/IKBKE-mediated phosphorylation of IRF3 [17], [18].

Here, we demonstrate that pharmacological inhibition of GSK3 inhibited NF-κB-mediated inflammation in response to viral infection, but more importantly that this inhibition extends to IRF3-mediated antiviral responses. Indeed, we confirm mechanistically that the inhibition is mediated through CTNNB1 since GSK3 inhibitor treatment, which induces accumulation of active CTNNB1 and abrogates antiviral responses, has almost no effect in CTNNB1 knockdown cells. The effect of CTNNB1 could be mediated directly through an interaction with IRF3 and p65 subunit of NF-κB as observed in our co-immunoprecipitation experiments (Figures 7 and S12). On the basis of these observations, we propose a novel role of a canonical-like WNT/CTNNB1 signaling pathway in a feedback inhibition of the antiviral innate immune response through regulation of IRF3- and NF-κB-dependent transcription (see Figure 8).

To our knowledge, there has been no comprehensive study describing a regulation of *IFNB1* expression by a canonical-like WNT signaling pathway. More importantly, we demonstrated for the first time the feedback inhibition of the RLR/MAVS/IRF3-mediated antiviral innate response by activation of WNT/CTNNB1 pathway induced upon viral infection. Our observations, combined with previously described role of mouse Wnt2 [35], Wnt3a [62] and *Drosophila melanogaster* WntD [34] in inflammatory response to bacterial infection, support a novel role of a canonical-like WNT pathway in the regulation of innate immunity to control excessive response to pathogens, including virus infection as newly reported in this study. The sheer diversity of the 19 WNT proteins and the 10 FZD receptors suggests that other WNT genes could serve as pathogen specific regulators of innate immunity. Different WNT and FZD expression profiles in various cell types could control the specificity of WNT-mediated signaling, a mechanism that would be reminiscent of the extracellular pathogen sensing by TLRs [63]. In addition, WNT signaling present the advantage of being limited to the vicinity of the infection site, since WNT posttranslational modification by lipid adducts allows them to stick tightly to cells membranes and the extracellular matrix, limiting their diffusion to lateral spreading between adjacent cells [64], [65].

In summary, our genome-wide RNAi screen identified 114 highly prioritized human genes potentially regulating virus-induced *IFNB1* expression. These genes acting either as positive or negative regulators upon silencing provide a functional validation of our RNAi data and identify novel regulators of *IFNB1* expression required to control virus infection and to prevent excessive activation in autoimmune diseases. Many genes of the WNT pathway (*WNT2B*, *WNT9B*, *WLS* and *CTNNB1*) are identified as negative regulators of RLR-mediated antiviral response upon gene silencing. WNT2B and WNT9B secretion in response to viral infection induce a CTNNB1-dependent signaling pathway leading to decreased magnitude of IFNB1 production and effector response in a feedback inhibition mechanism. Exploiting the actions of human encoded regulators of the innate immune response by developing immunomodulatory molecules presents an alternative strategy with a potential to treat a broad range of viral infections. In particular, the discovery of drugs that target canonical-like WNT pathways may be therapeutically useful for limiting viral infection or virus-induced inflammation.

## Materials and Methods

### Ethics statement

Primary human hepatocytes were purified by collagenase perfusion from normal biopsies obtained from healthy peritumoral liver tissues of patients undergoing partial hepatectomy for hepatic tumors [66]. Wedge samples (20–50 g) were flushed of blood and maintained overnight in a preservation solution (Belzer UW; Dupont Pharma) at 4°C. At time of perfusion, a selected vein was cannulated with a tip to allow circulation of the buffers in the whole liver sample. All other surrounding veins were subsequently sealed with Vetbound veterinary glue (3M) to allow good dispersion of the buffers and increase the pressure in the whole liver sample. Perfusion starts with calcium free HEPES buffer for 15 min at 20 ml/min following perfusion with 0.55 mg/ml type 2 collagenase (Worthington Biochemical Corporation) and 1.65 mg/ml CaCl2 for 30 min at 15 ml/min. At the end, liver was opened and cells washed in L-15 media. Viability was assessed by trypan blue exclusion. Cells were seed at a 50 000 cells/cm2 with William's E media supplemented with 10% FBS. All procedures followed were in accordance with the ethical commission of the CHUM, Hôpital St-Luc, Montréal, Canada and with the Helsinki Declaration. Human monocyte-derived macrophages (MDM) were prepared from peripheral blood mononuclear cells (PBMC) purified from whole blood of healthy donors by standard Ficoll (GE Sciences) gradient centrifugation. Monocytes were obtained from PBMC by plastics adherence (>97% CD14+). Following extensive washes to remove lymphocytic non-adherent cells, adherent monocytes were allowed to differentiate into MDM for 5 days in the presence of 50 ng/mL granulocyte-monocyte colony stimulating factor (M-CSF, R&D). The study was approved by the IRCM Research Ethic Review Board and in accordance with the Helsinki Declaration. Samples were collected after written informed consent had been obtained.

### Expression vectors

*WNT2B*, *WNT9B*, *CTNNB1* cDNA were obtained from Open Biosystems. Following PCR-amplification, PCR products were cloned into pcDNA3.1-Hygro-MCS using EcoRV/HindIII [67]. All constructs were verified by nucleotide sequencing. pIFNB1-LUC, pISG56-LUC and p2xNF-κB-LUC luciferase reporter constructs were previously described [68]–[70]. To generate stable 293T cells harboring the pIFNB1-LUC promoter, we replaced the MluI-BamHI fragment encompassing the CMV promoter of pcDNA3.1-hygro(+) with the MluI-BamHI fragment of IFNβ-pGL3 [69], which includes two copy of the *IFNB1* promoter (−280/+20) and the firefly luciferase coding sequence. pEF1α-LUC was generated after PCR amplification of the EF1α promoter from pEF/JFH1-Rz/Neo [71] and cloning into pcDNA3.1_fLUC_hygro(+) using MluI and BamHI. HCV genotype 2a strain JFH1 p7(+) [71] and J6/JFH-1(p7-Rluc2A) [72] were previously described.

### Cells lines and culture

HEK 293T (human embryonic kidney), Huh7 (human hepatoma) and HeLa (human epithelial carcinoma) cell lines were cultured in Dulbecco's modified Eagle's medium (DMEM, Wisent). A549 (human lung adenocarcinoma epithelial) were cultured in Ham's F-12 medium (Invitrogen). Both media were supplemented with 10% fetal bovine serum, 100 units/ml penicillin, 100 µg/ml streptomycin and 2 mM glutamine (all from Wisent). Normal human bronchial epithelial cells (NHBE) were obtained from Lonza, cultured in BEGM medium (Lonza) and used between passage 2 and 3. Cell populations of HEK 293T stably harbouring the pIFNB1-LUC and pEF1α-LUC used in the screens were produced after selection with 200 µg/ml of hygromycin B (Wisent). Transient transfections were performed with linear 25 kDa polyethylenimine (PEI) (Polysciences, Inc) at a 3 µg PEI to 1 µg DNA ratio for all plasmids and with lipofectamine 2000 (invitrogen) for polyI∶C. siRNA ON-TARGETplus SMARTpool, Human CTNNB1 (1499) (Thermo Scientific) was transfected with lipofectamine RNAi max (Invitrogen) according to the reverse transfection manufacturer protocol for 72 hours. GSK3 inhibitors BIO (GSK3 inhibitor IX, Sigma) and BIO-acetoxime (GSK3 inhibitor X, EMD Millipore) and inhibitor of TCF/LEF interaction PNU74654 (TOCRIS) were added for 16 hours at the indicated concentrations in 0.5% DMSO.

### Lentiviral shRNA library production

MISSION TRC shRNA lentiviral library containing _∼_75,000 individual clones representing _∼_15,000 genes was purchased from Sigma-Aldrich. Three individual clones per gene were chosen for lentiviral production, covering different regions of the gene sequence. For the genome-wide production of lentiviral particles, HEK293 cells (2×10^4^) were plated one day before the transfection. All transfections were performed using a Biomek FX (Beckman Coulter) enclosed in a class II cabinet according to MISSION Lentiviral Packaging Mix protocol (SHP001). As control the MISSION shRNA NT clone (Sigma SHC002) was included in each 96-well plates. The lentivirus-containing supernatants were collected at 24 and 48 hours post-transfection and pooled. NT shRNA controls and 4% of random samples of each plate were used to measure lentiviral titers for quality control purposes. Titers were determined by limiting dilution assay using HeLa cells. The samples (20 µl) were serially diluted in complete DMEM containing 8 µg/ml of hexadimethrine bromide (Polybrene from Sigma). Dilutions (1∶400 or 1∶10,000) were added to preparation of HeLa cells and media were changed at day 3 and 5 with complete DMEM containing 1 µg/ml of puromycin (Wisent). After four days with the selective agent, cells were stained with 1.25% crystal violet and counted in plaque-forming units (PFU) allowing the lentiviral titer determination.

### Genome-wide shRNA screen

Cells were seeded in white 96-well plates at a density of 5,000 293T pIFNB1_LUC and 1,250 293T pEF1α-LUC in 100 µl of complete phenol-red free DMEM containing 4 µg/ml polybrene. Infection with lentivirus encoding shRNA (pool of three shRNAs for the primary screen and five individual shRNAs for secondary screens) were carried out immediately after cell seeding at a multiplicity of infection (MOI) of 5 and incubated for four days at 37°C in an atmosphere of 5% CO_2_. Cells were infected with 100 HAU/ml of SeV (Cantell Strain, Charles River Labs) for 16 hours before cell lysis and firefly luminescence reading in 100 mM Tris acetate, 20 mM Mg acetate, 2 mM EGTA, 3.6 mM ATP, 1% Brij 58, 0.7% β-mercaptoethanol and 45 µg/ml luciferine pH 7.9 buffer. All infections were performed using a Biomek FX (Beckman Coulter) enclosed in a class II cabinet.

### Large-scale lentiviral shRNA production

293T cells were transfected using PEI with 6 µg pLKO.1-puro encoding shRNA targeting WNT2B (TRCN0000033376 and TRCN0000033377), WNT9B (TRCN0000062069 and TRCN0000062070), WLS (TRCN0000133858 and TRCN0000133999), CTNNB1 (TRCN0000003843, TRCN0000003844 and TRCN0000003845) or shRNA non-target (NT, Sigma), 1.5 µg pMDLg/pRRE, 1.5 µg pRSV-REV and 3 µg pVSVg as previously described [73]. After 48 hours, medium containing lentiviral vector was harvested and cellular debris were removed by filtration (0.45 µm). Titers were determined by limiting dilution assay using HeLa cells, as described above.

### High-throughput qRT-PCR assay

For the shRNA validation screen, total RNA was extracted in 96-well plate format using “RNAqueous – 96 automated” kit (Ambion) with a Multimek-96 (Beckman Coulter). RNA concentrations were measured using Ribogreen reagent (Invitrogen) on the Envision plate reader (Perkin Elmer). After normalisation of RNA at 200 ng/well, reverse transcription reactions were performed with a High Capacity cDNA Reverse Transcription kit (Applied Biosystems). Quantitative PCR assays were designed following the Universal Probes Library (UPL) from Roche. The best primer/probe combinations were calculated online with the ProbeFinder assay design software. qPCR reactions were performed in 384-well plates using 2 µl of cDNA samples, 5 µl of the Light Cycler 480 Probes Master (Roche), 1 µM of gene specific primers (IDT) and 2 µM of the matching UPL probe in a total of 10 µl (supplementary Table 3 for the complete list of primers and probes used). Amplification conditions on the Light Cycler 480 (Roche) for the UPL were initial denaturation of 5 minutes at 95°C followed by 45 cycles of 10 seconds at 95°C, 30 seconds at 60°C and 1 second at 72°C. All qPCR were run in duplicates and the relative quantification of mRNAs from the *IFNB1* and shRNA targeted genes was determined with the average of Ct (cycle threshold) using the ΔΔCt method. Briefly, the difference in the cycle threshold (ΔCt) value was determined by subtracting the Ct value for the transcripts of interest, from the Ct value of the endogenous control (*ACTB*: β-actin) and compared with a shRNA NT calibrator: ΔΔCT = ΔCt_Sample_−ΔCt_Calibrator_. Relative expression (RQ) was calculated using the Sequence Detection System (SDS) 2.2.2 software (Applied Biosystems) and the formula RQ = 2^−ΔΔCT^. A second set of primers and probe were designed for genes for which no amplification was obtained in the first round of qPCR (supplementary Table 3). A not determined (nd) note is indicated when no results were available after two rounds of qPCR (supplementary Table 1).

### Large-scale RNA extraction and qRT-PCR

Total cellular RNA was extracted with the RNeasy Mini kit (Qiagen). Reverse transcription was performed on 500 ng total cellular RNA using the High Capacity cDNA Reverse Transcription kit (Applied Biosystems, see (supplementary Table 4 for the list of primers and probes). PCR reactions were performed using 1.5 µl of cDNA samples (15 ng), 5 µl of the Fast TaqMan PCR Master Mix (Applied Biosystems), 10 pmol of each primer (IDT) and 5 pmol of the UPL probe (Roche) in a total volume of 10 µl. The ABI PRISM 7900HT Sequence Detection System (Applied Biosystems) was used to detect the amplification level and was programmed to an initial step of 3 minutes at 95°C, followed by 40 cycles of 5 seconds at 95°C, 30 seconds at 60°C and 1 second at 72°C. All reactions were run in triplicate on biological duplicate and the average values were used for quantification. *ACTIN* and *HPRT1* (hypoxanthine phosphoribosyltransferase 1) were used as endogenous controls. The relative quantification of target genes was determined by using the ΔΔCt method, as described above.

### Immunofluorescence analysis

HEK 293T cells were seeded in cover slip-containing 24-well plates. Twenty-four hours later, cells were infected with SeV for 16 hours before being washed twice with PBS, fixed with 4% paraformaldehyde-containing PBS during 20 minutes at room temperature and then permeabilized in 0.2% Triton X-100/PBS during 15 minutes. Blocking was made in PBS with 10% normal goat serum, 5% bovine serum albumin (BSA) and 0.02% sodium azide during 45 minutes at room temperature. Following three rapid washes, cells were labelled with mouse anti-CTNNB1 active (Millipore) and rabbit anti-IRF3 (IBL) primary antibodies diluted in 5% BSA/0.02% sodium azide/PBS during 2 hours. Slides were washed three times in PBS and then labelled with anti-mouse AlexaFluor 488 and anti-rabbit AlexaFluor 546 secondary antibodies (Invitrogen) diluted in 5% BSA/0.02% sodium azide/PBS during 1 hour. Cells were extensively washed and incubated with Hoechst dye (Invitrogen) at a final concentration of 1 µg/mL in PBS. Following three rapid washes, slides were mounted using DABCO (Sigma-Aldrich) as an anti-fading agent. Labelled cells were then examined by laser scanning microscopy using a LSM510 confocal microscope (Zeiss).

### Microarray analysis

The microarray studies were performed with HEK 293T cells infected for 16 hours with SeV (100 HAU/ml) or treated for 16 hours with a mixture of IFN-α from human leukocytes (400 U/ml; Sigma) and with untreated cells as control. A total of 10 µg of RNA was reverse transcribed using oligo(dT) 16–18 primers and SuperScript II Reverse Transcriptase (Invitrogen) according to the manufacturer's instructions. Following purification using QIAquick PCR Purification kit (Qiagen), up to 1 µg of purified cDNA was mixed with 5′-Cy3 labeled random nonamers (Trilink Biotechnology) and heated at 95°C for 10 minutes and transferred on ice for 10 minutes. Samples were mixed with 1 mM dNTP and 2 µl of 3′-5′ exo-Klenow fragment (New England Biolabs) and incubated at 37°C for 2 hours. The labeling reaction was stopped using 50 µM EDTA and the DNA precipitated using 0.5 M NaCl and 1 volume isopropanol, washed with 80% ethanol and resuspended in water. Hybridizations were carried out using the Human GE 4×44K v2 Microarrays (Agilent Technologies) containing probes targeting 27,958 Entrez Gene RNAs. Arrays were scanned at 5 µm resolution using a GenePix4000B scanner (Molecular Devices). Data from scanned images were extracted using GenePix 6.1 (Axon) and processed and normalized using ArrayPipe (v2.0). Processed data was used as input for linear modeling using Bioconductor's limma package, which estimates the fold-change between predefined groups by fitting a linear model and using an empirical Bayes method to moderate standard errors of the estimated log-fold changes in expression values from each probe set. *P* values from the resulting comparison were adjusted for multiple testing according to the method of Benjamini and Hochberg.

### Western immunoblot analysis

Cells were washed twice with ice-cold phosphate-buffered saline (PBS; Wisent), harvested and lysed in 10 mM Tris-HCl, 100 mM NaCl, 0.5% Triton X-100, pH 7.6 with EDTA-free Protease Inhibitor Cocktail (Roche). Cell lysates were clarified by centrifugation at 13,000 g for 20 min at 4°C and subjected to sodium dodecyl sulfate-polyacrylamide gel (SDS-PAGE). Western blot analysis was performed using mouse anti-WNT2B (Santa Cruz), anti-ACTIN (Chemicon International), anti-CTNNB1 active (Millipore), anti-FLAG (Sigma), anti-IRF3 (Santa Cruz), anti-NS3 (HCV, Abcam), anti-p65 (Santa Cruz) and rabbit anti-CTNNB1 total (Epitomics), anti-CTNNB1-P-Ser552 (Cell Signaling), anti-IFIT2 (Novus Biologicals), anti-IFIT1 (Novus Biologicals), anti-SeV (HN) [first described by Ilkka Julkunen 74], anti-WNT9B (Aviva systems biology) and anti-NFKBIA-P-Ser32 (Cell Signaling). HRP-conjugated secondary antibodies were from Bio-Rad. The chemiluminescence reaction was performed using the Western Lighting Chemiluminescence Reagent Plus (PerkinElmer).

### Luciferase assay

293T cell population stably expressing pIFNB1-LUC were cultured in white opaque 96-well plates with complete phenol red-free DMEM and transfected with 1 ng of Rluc expressing plasmid used to normalized the firefly luciferase readout. For the other fLUC reporter constructs, 293T cells were transfected with 20 ng of M50 Super 8×TOP-flash [40] (Addgene plasmid 12456), pIFNB1-LUC, pISG56-LUC or p2xNF-κB-LUC and 1 ng of Rluc. Cells were lysed directly and luciferase activities were determined by a dual-luciferase reporter assay system (Promega).

### SeV, HCV and IFN-α and purified WNT stimulations

Cells were infected with 100 HAU/ml of Sendai virus (Cantell Strain, Charles River Labs) or stimulated with mixture of IFN-α from human leukocytes (400 U/ml; Sigma) for 4 hours in A549 cells and MDM, 5 hours in human primary hepatocytes and 16 hours in 293T, Huh7 and HeLa cells before luminescence reading, immunoblot analysis or qRT-PCR. Primary human hepatocytes were infected for 48 hours with HCV genotype 2a strain JFH1 p7(+) and Huh7 cells were infected for 96 hours with J6/JFH-1(p7-Rluc2A). HCV particles were produced as previously described [43]. Purified WNT9B and WNT3A proteins (R&D systems) were reconstituted at 100 µg/ml in PBS containing 0.1% BSA and added to SeV-infected HEK 293T cell supernatant for 16 hours.

### IFN-β ELISA

Secreted IFN-β protein quantification was measure with *VeriKine* Human IFN-α Serum Sample ELISA Kit (PBL Interferon Source) using 50 µl of HEK 293T cells supernatant previously transduced with lentivirus-expressing shRNAs for three days before infection with SeV for 16 hours.

### Statistical analyses

Data are presented as the mean ± standard error of the mean (SEM). Statistical significance for comparison of two means was assessed by an unpaired Student's t test. Analyses were performed using the Prism 5 software (GraphPad). Statistical relevance was evaluated using the following *p* values: *p*<0.05 (*), *p*<0.01 (**) or *p*<0.001 (***).

## Supporting Information

Figure S1 **Statistical analysis of the genome-wide gene silencing study of virus-induced innate immune responses.** (A) Average luminescence determination of control shRNAs (NT, MAVS_45 and NLRX1_46) on *IFNB1* promoter-driven luciferase activity that were incorporated in each 96-well plate of the genome-wide screen. (B) Gaussian distribution of all individual *IFNB1* promoter-driven luciferase data points of the genome-wide screen in percentage inhibition of control shRNA NT. (C) Individual *IFNB1* promoter-driven luciferase data points of shRNA targeting 15,357 human genes in percentage inhibition of control shRNA NT. (D) Coefficient of variation (CV) of each 96-well plate in the primary screen. Dotted lines delimitate six different screening campaigns (run 1–6) for completion of the primary screen. The average CV for each screening campaign is indicated (green). (E) Number of hits selected per tested plate using the strictly standardized mean difference (SSMD) as a cutoff (SSMD≤−1.314 and SSMD≥1.662). Dotted lines delimitate six different screening campaigns (run 1–6) for completion of the primary screen. The average hit rate for each screening campaign is indicated (blue).
(TIF)

Figure S2 **Functional profiling data of the 114 gene hits identified as potential regulators of antiviral innate responses and classified within the four functional groups.** Data are represented as heat map indicating log_2_ effect of silencing each gene hit in secondary assays. Positive regulators are depicted in blue and negative regulators are depicted in yellow. Manual clustering was performed for each gene hit confirmed with at least two shRNAs in SeV confirmation screen and validated with endogenous *IFNB1* mRNA quantification by classification into one of the four functional groups: (A) SeV specific; (B) Cytoplasmic dsRNA sensing; (C) MAVS-dependent signaling; (D) Nuclear import or transcription factor-dependent process.
(TIF)

Figure S3 **Validation of the knockdown efficiency of shRNAs and siRNAs used in this study.** (A–D) *WNT2B* (A), *WNT9B* (B), *WLS* (C) and *CTNNB1* (D) mRNA levels in HEK 293T cells transduced with two-independent shRNAs per gene for four days. qRT-PCR determination represents the average mRNA RQ normalized versus ACTIN and HPRT1 mRNA.
(TIF)

Figure S4 **WNT2B and WNT9B ligands as novel negative regulators of antiviral innate immunity.** Immunoblot analysis of WNT9B and IFIT1 (top), and qRT-PCR determination of *WNT2B*, *IFNB1*, *IFIT1* and *TNF* mRNA levels (bottom) in WNT2B or WNT9B knockdown HEK 293T cells following infection with SeV. Heat map data are log_2_ scale of the average mRNA RQ normalized versus ACTIN and HPRT1 mRNA.
(TIF)

Figure S5 **WNT2B and WNT9B knockdown phenotype can be rescued by expression of the corresponding cDNA harboring silent mutations to render it resistant to RNAi degradation.** Fold induction of *IFNB1* promoter-driven luciferase activity in WNT2B and WNT9B knockdown HEK 293T cells following infection with SeV for 16 hours transfection. Knockdown cells were transfected with an empty expression vector (pcDNA3.1) or WNT2B and WNT9B immune to RNAi expression vectors for 48 hours. *P* values<0.05 (*) are indicated.
(TIF)

Figure S6 **WNT2B and WNT9B are not induced following SeV infection or IFN-α treatment.** (A–D) Kinetic studies of SeV infection and IFN-α treatment on gene transcription of *WNT2B* (A), *WNT9B* (B), and representative effector genes *IFIT1* (C) and *IFNB1* (D) by qRT-PCR quantification of mRNA levels in HEK 293T cells.
(TIF)

Figure S7 **WNT2B and WNT9B secretion in various cell lines and in WLS knockdown cells.** (A) Immunoblot analysis of WNT2B secretion in supernatants of HEK 293T, HeLa and Huh7 cells following a 16 hours SeV infection or IFN-α treatment. (B) Immunoblot analysis of WNT2B and WNT9B secretion in supernatants of HEK 293T treated with shRNA targeting WLS for four days and subjected to IFN-α treatment for 8 hours.
(TIF)

Figure S8 **WNT2B and WNT9B knockdown phenotype on innate antiviral immunity are additive.** Fold induction of *IFNB1* promoter-driven luciferase activity in single or double knockdown WNT2B, WNT9B and CTNNB1 HEK 293T cells following infection with SeV for 16 hours. Cells were transduced with a mixture of shRNA NT (MOI = 5) and targeted gene shRNA (MOI = 5) for single knockdown and a mixture of two targeted gene shRNAs (MOI = 5 for each one) for double knockdown, to maintain a MOI = 10 in all conditions. *P* values<0.01 (**) or <0.001 (***) are indicated.
(TIF)

Figure S9 **SeV infection induces CTNNB1 nuclear translocation.** Confocal analysis of A549 cells using Hoechst, anti-CTNNB1 active form and anti-p65 antibodies without virus infection or following 6 hours infection with SeV.
(TIF)

Figure S10 **Effect of GSK3 inhibition on transcriptional activity of representative genes of innate immunity in CTNNB1 knockdown HEK 293T cells - Immunoblot analysis of CTNNB1 knockdown and GSK3 inhibitor-treated A549 cells.** (A–C) qRT-PCR quantification of *IFNB1* (A), *IFIT1* (B) and *TNF* (C) mRNA levels in HEK 293T transduced with lentivirus-expressing shRNA NT (control) or shRNA 45 targeting *CTNNB1* for four days and subjected to SeV infection and treatment with GSK3 inhibitor BIO (5 µM) for 16 hours. qRT-PCR determination represents the average mRNA RQ normalized versus ACTIN and HPRT1 mRNA. (D) Immunoblot analysis of dephosphorylated active CTNNB1 at Ser37/Thr41 and IFIT1 in A549 cells transduced with lentivirus-expressing shRNA NT (control) or shRNA 45 targeting *CTNNB1* for four days and subjected to treatment with GSK3 inhibitors BIO (5 µM) or BIO-acetoxime (10 µM) and SeV infection for 6 hours.
(TIF)

Figure S11 **WNT/GSK3/CTNNB1 signaling acts as a negative regulator of innate immune response in primary human hepatocytes infected with SeV.** Fold induction of *IFIT1* (A) and *TNF* (B) mRNAs in primary human hepatocytes transduced with lentivirus-expressing shRNA NT, shRNA WNT9B 70 or shRNA CTNNB1 44 for four days and subjected SeV infection for 5 hours. qRT-PCR determination represents the average mRNA RQ normalized versus ACTIN and HPRT1 mRNA. *P* values<0.01 (**) or <0.001 (***) are indicated.
(TIF)

Figure S12 **CTNNB1 associates with IRF3 and NF-κB subunit p65.** (A) HEK 293T cells were co-transfected with CTNNB1 and FLAG-MCS (control), FLAG-eYFP (control), FLAG-IRF3, FLAG-IRF3(5D) p65-FLAG or TBK1-FLAG expressing plasmids for forty-eight hours. Cell extracts were prepared following infection with SeV for 16 hours before being subjected to immunoprecipitation directed against FLAG. Immune complexes were analyzed by Western blotting using anti-FLAG and anti-CTNNB1 antibodies. (B) HEK 293T cells were transfected with FLAG-eYFP (control) or FLAG-CTNNB1 expressing plasmids for forty-eight hours. Cell extracts were prepared following 16 hours of treatment with GSK3 inhibitor BIO (5 µM), SeV infection or both before being subjected to immunoprecipitation directed against FLAG. Immune complexes were analyzed by Western blotting using anti-FLAG and anti-p65 and anti-IRF3 antibodies.
(TIF)

Table S1 **Summary of gene silencing screening data.**
(XLSX)

Table S2 **Venn diagram overlaps.** Gene symbols identified with a (*) were validated in secondary screens, but discarded in the pEF1α specificity assay.
(XLSX)

Table S3 **Oligonucleotide sequences and UPL probe used for HTS qRT-PCR.**
(XLSX)

Table S4 **Oligonucleotide sequences and UPL probe used for qRT-PCR.**
(XLSX)
