# Supplementary figures and images for: Genome-wide RNAi Screen Reveals a New Role of a WNT/CTNNB1 Signaling Pathway as Negative Regulator of Virus-induced Innate Immune Responses
Source: PLoS Pathog. 2013 Jun 13;9(6):e1003416. doi: 10.1371/journal.ppat.1003416 (PMC3681753; doi:10.1371/journal.ppat.1003416)

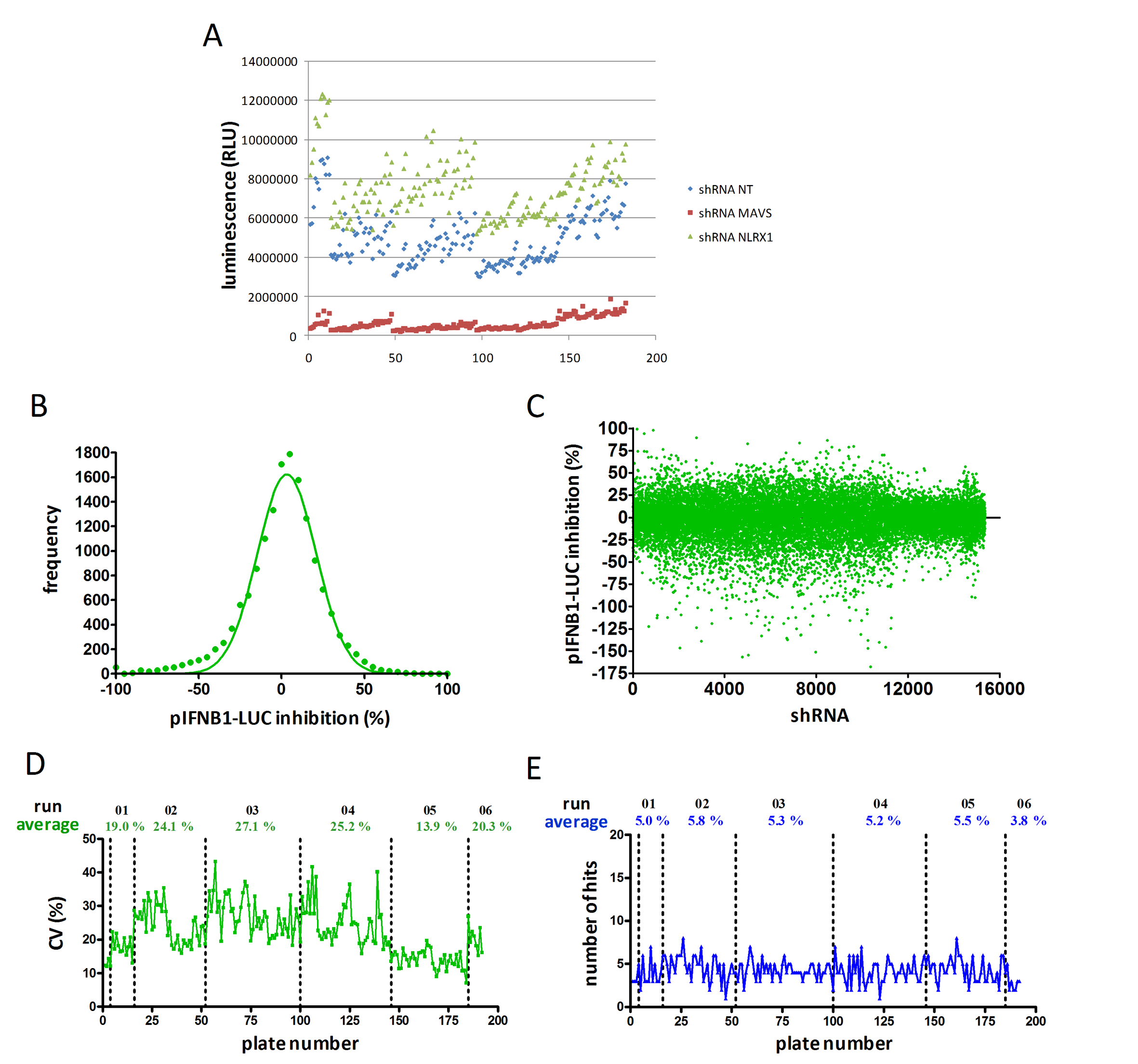

Supplement: Figure S1 — Statistical analysis of the genome-wide gene silencing study of virus-induced innate immune responses. (A) Average luminescence determination of control shRNAs (NT, MAVS_45 and NLRX1_46) on IFNB1 promoter-driven luciferase activity that were incorporated in each 96-well plate of the genome-wide screen. (B) Gaussian distribution of all individual IFNB1 promoter-driven luciferase data points of the genome-wide screen in percentage inhibition of control shRNA NT. (C) Individual IFNB1 promoter-driven luciferase data points of shRNA targeting 15,357 human genes in percentage inhibition of control shRNA NT. (D) Coefficient of variation (CV) of each 96-well plate in the primary screen. Dotted lines delimitate six different screening campaigns (run 1–6) for completion of the primary screen. The average CV for each screening campaign is indicated (green). (E) Number of hits selected per tested plate using the strictly standardized mean difference (SSMD) as a cutoff (SSMD≤−1.314 and SSMD≥1.662). Dotted lines delimitate six different screening campaigns (run 1–6) for completion of the primary screen. The average hit rate for each screening campaign is indicated (blue). (TIF) [file ppat.1003416.s001.tif]

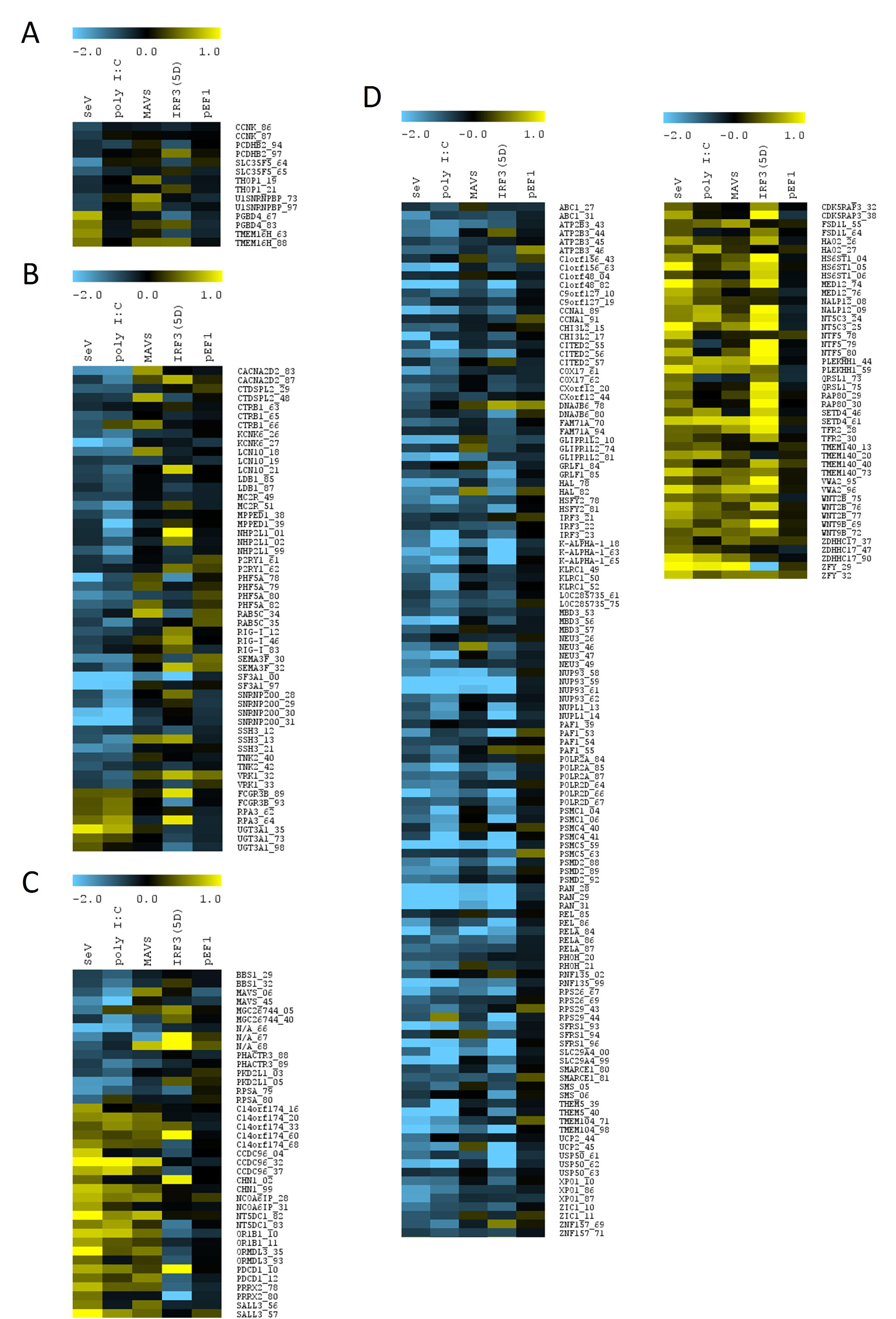

Supplement: Figure S2 — Functional profiling data of the 114 gene hits identified as potential regulators of antiviral innate responses and classified within the four functional groups. Data are represented as heat map indicating log2 effect of silencing each gene hit in secondary assays. Positive regulators are depicted in blue and negative regulators are depicted in yellow. Manual clustering was performed for each gene hit confirmed with at least two shRNAs in SeV confirmation screen and validated with endogenous IFNB1 mRNA quantification by classification into one of the four functional groups: (A) SeV specific; (B) Cytoplasmic dsRNA sensing; (C) MAVS-dependent signaling; (D) Nuclear import or transcription factor-dependent process. (TIF) [file ppat.1003416.s002.tif]

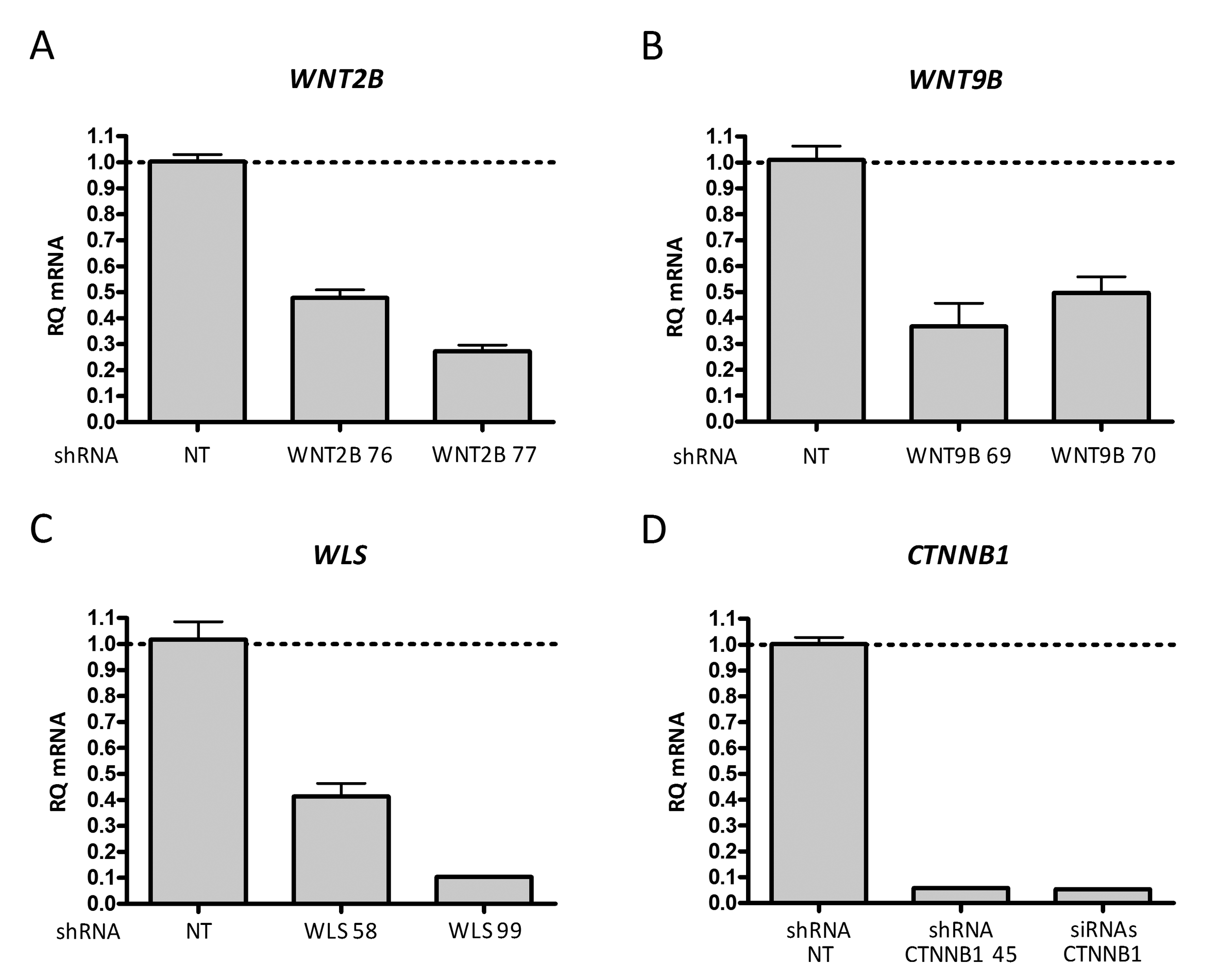

Supplement: Figure S3 — Validation of the knockdown efficiency of shRNAs and siRNAs used in this study. (A–D) WNT2B (A), WNT9B (B), WLS (C) and CTNNB1 (D) mRNA levels in HEK 293T cells transduced with two-independent shRNAs per gene for four days. qRT-PCR determination represents the average mRNA RQ normalized versus ACTIN and HPRT1 mRNA. (TIF) [file ppat.1003416.s003.tif]

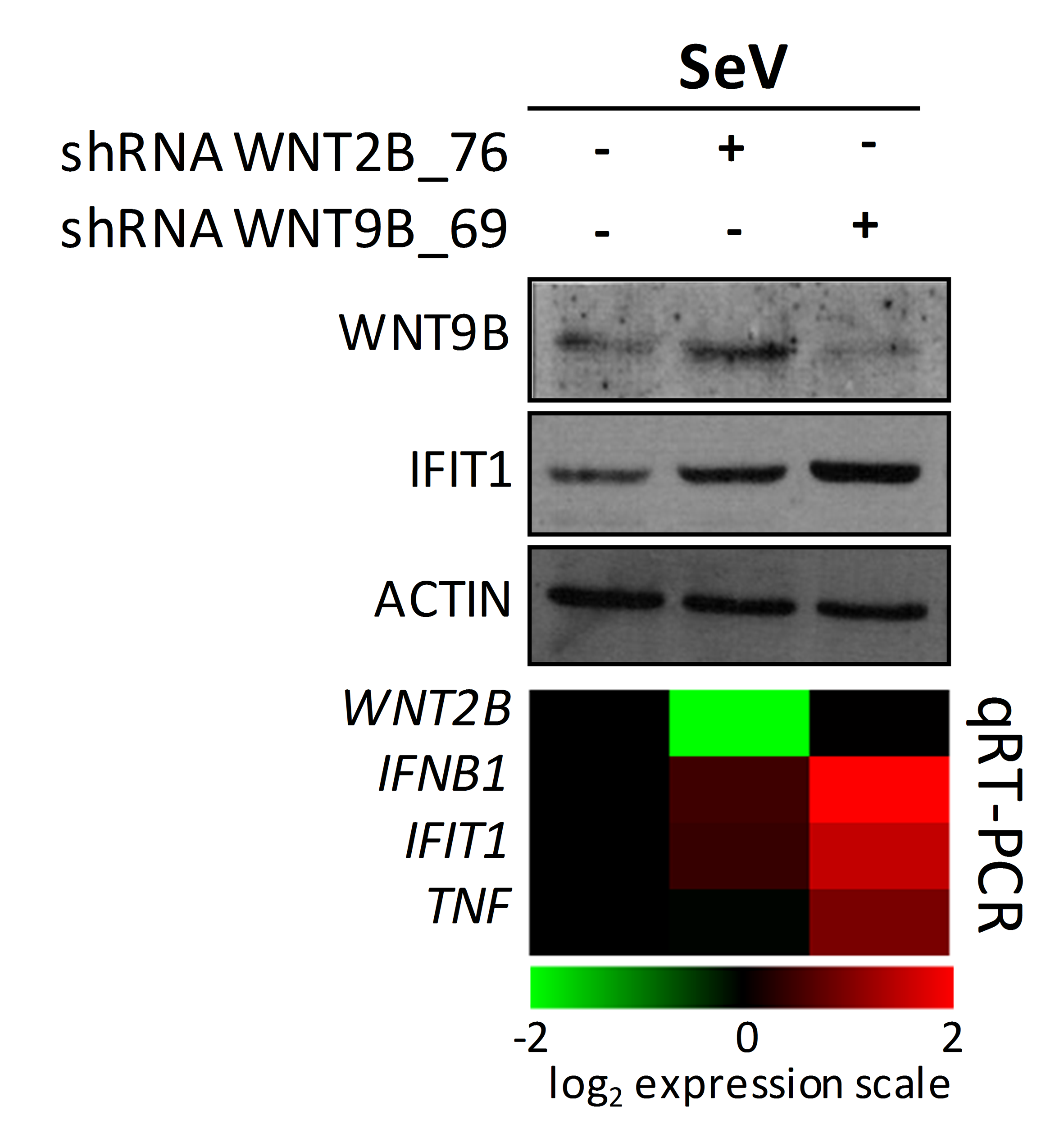

Supplement: Figure S4 — WNT2B and WNT9B ligands as novel negative regulators of antiviral innate immunity. Immunoblot analysis of WNT9B and IFIT1 (top), and qRT-PCR determination of WNT2B, IFNB1, IFIT1 and TNF mRNA levels (bottom) in WNT2B or WNT9B knockdown HEK 293T cells following infection with SeV. Heat map data are log2 scale of the average mRNA RQ normalized versus ACTIN and HPRT1 mRNA. (TIF) [file ppat.1003416.s004.tif]

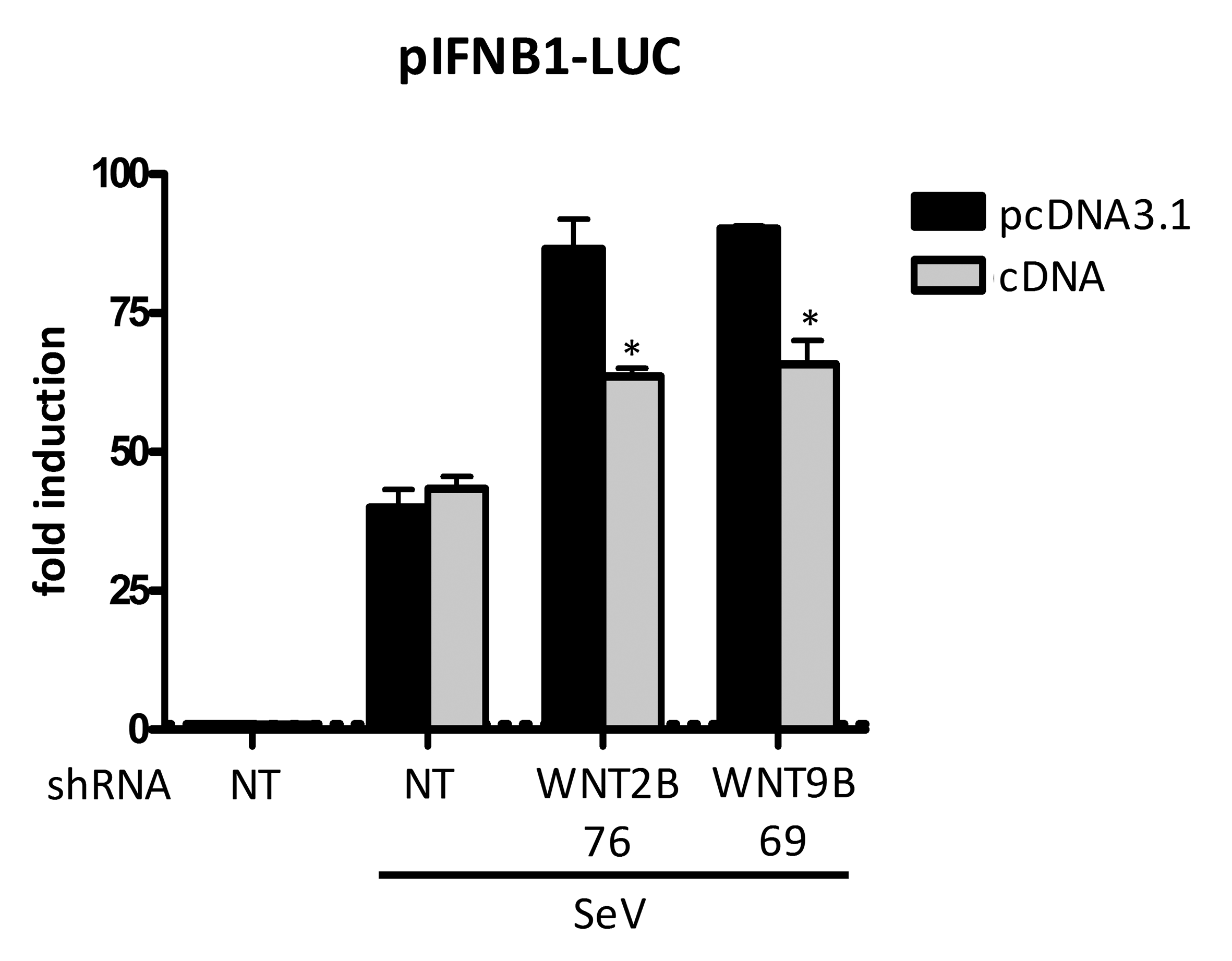

Supplement: Figure S5 — WNT2B and WNT9B knockdown phenotype can be rescued by expression of the corresponding cDNA harboring silent mutations to render it resistant to RNAi degradation. Fold induction of IFNB1 promoter-driven luciferase activity in WNT2B and WNT9B knockdown HEK 293T cells following infection with SeV for 16 hours transfection. Knockdown cells were transfected with an empty expression vector (pcDNA3.1) or WNT2B and WNT9B immune to RNAi expression vectors for 48 hours. P values<0.05 (*) are indicated. (TIF) [file ppat.1003416.s005.tif]

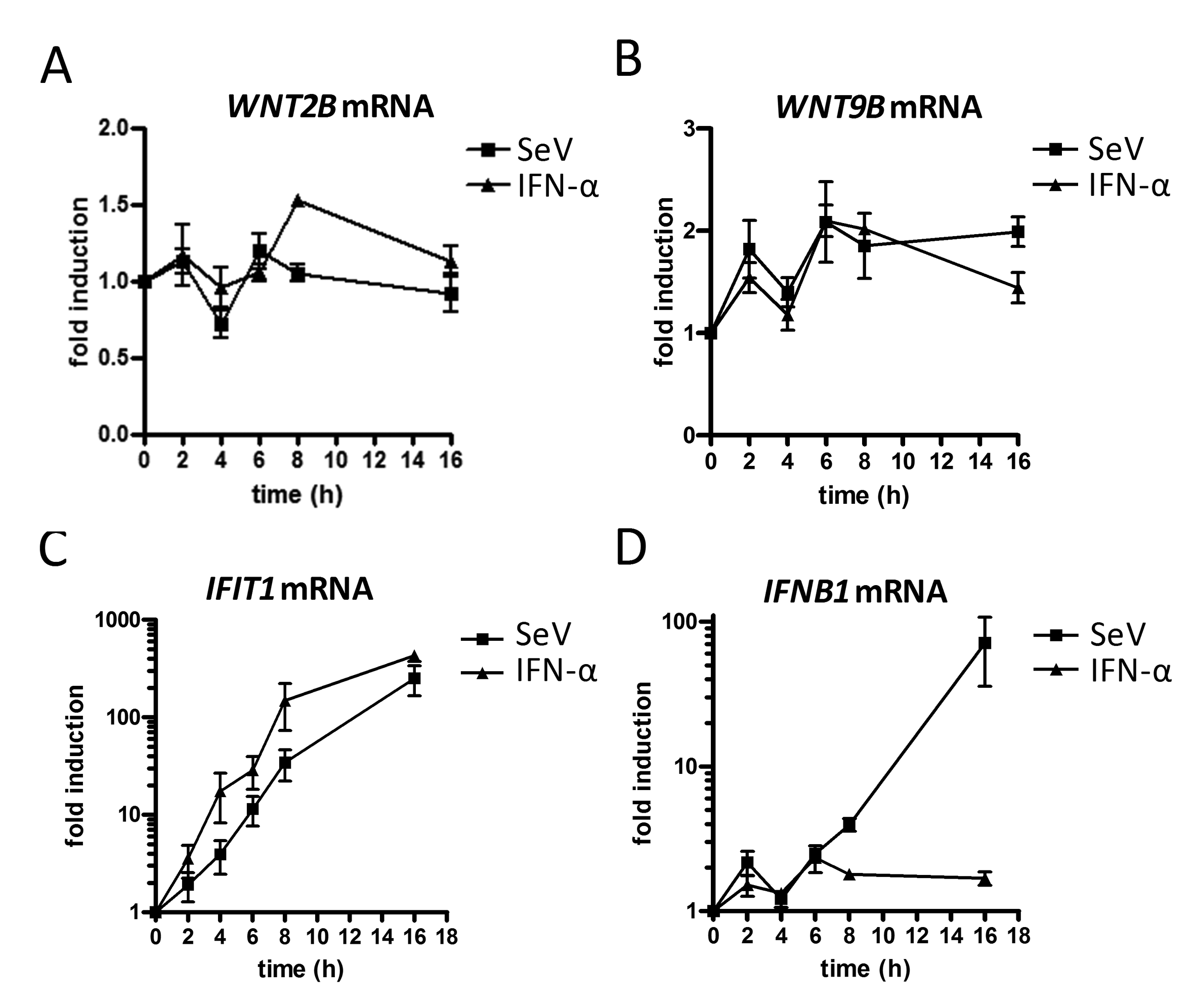

Supplement: Figure S6 — WNT2B and WNT9B are not induced following SeV infection or IFN-α treatment. (A–D) Kinetic studies of SeV infection and IFN-α treatment on gene transcription of WNT2B (A), WNT9B (B), and representative effector genes IFIT1 (C) and IFNB1 (D) by qRT-PCR quantification of mRNA levels in HEK 293T cells. (TIF) [file ppat.1003416.s006.tif]

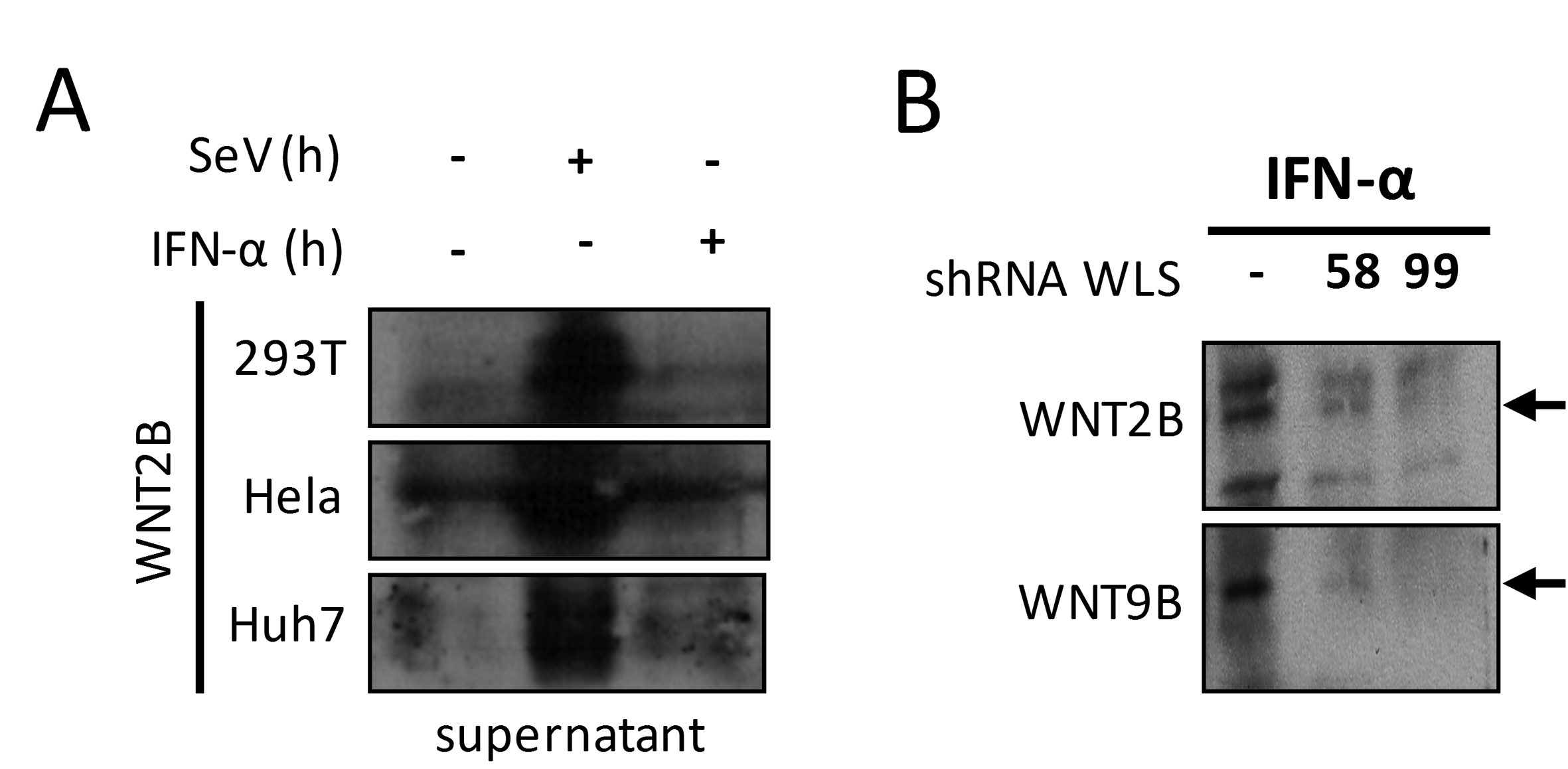

Supplement: Figure S7 — WNT2B and WNT9B secretion in various cell lines and in WLS knockdown cells. (A) Immunoblot analysis of WNT2B secretion in supernatants of HEK 293T, HeLa and Huh7 cells following a 16 hours SeV infection or IFN-α treatment. (B) Immunoblot analysis of WNT2B and WNT9B secretion in supernatants of HEK 293T treated with shRNA targeting WLS for four days and subjected to IFN-α treatment for 8 hours. (TIF) [file ppat.1003416.s007.tif]

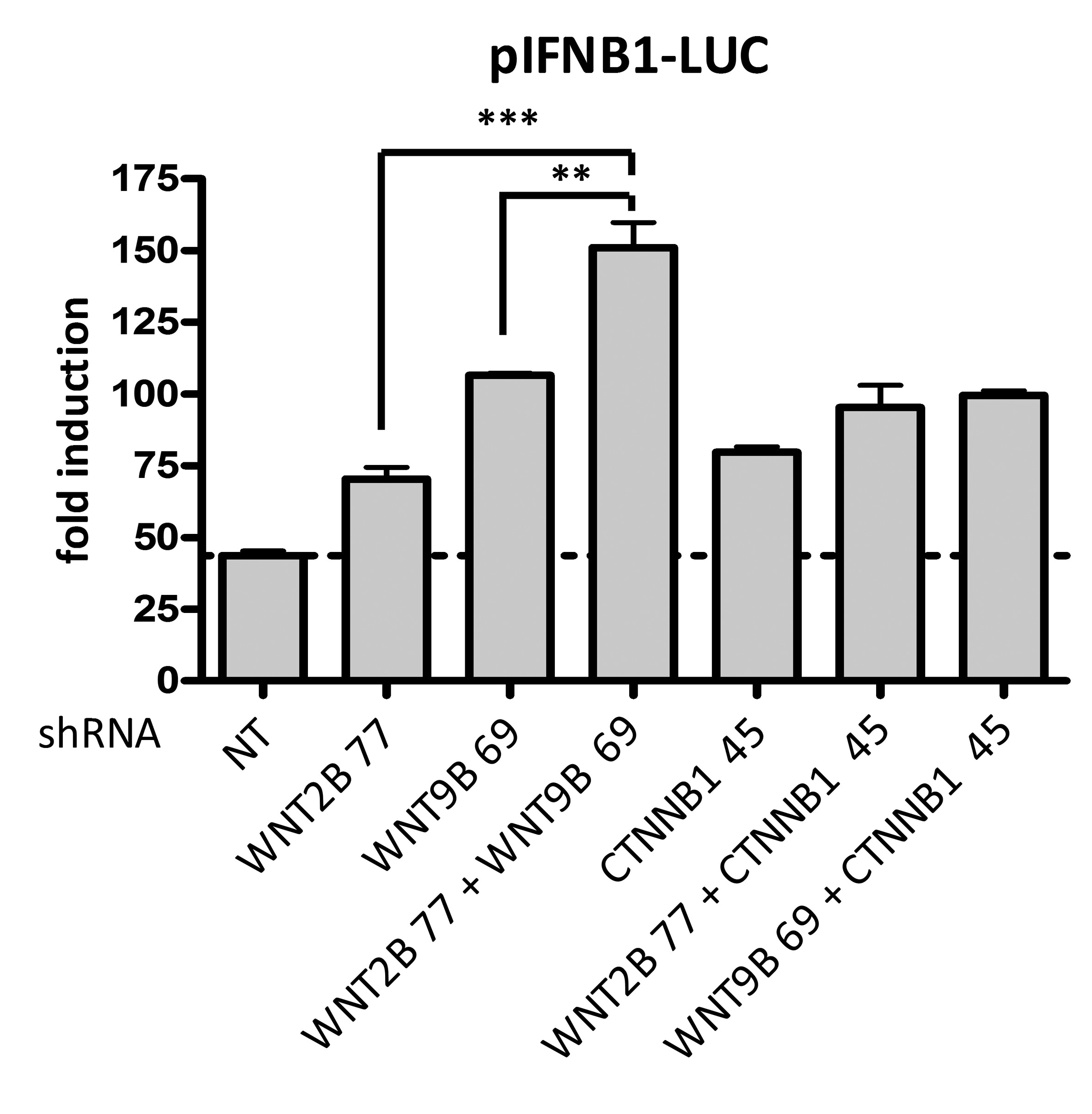

Supplement: Figure S8 — WNT2B and WNT9B knockdown phenotype on innate antiviral immunity are additive. Fold induction of IFNB1 promoter-driven luciferase activity in single or double knockdown WNT2B, WNT9B and CTNNB1 HEK 293T cells following infection with SeV for 16 hours. Cells were transduced with a mixture of shRNA NT (MOI = 5) and targeted gene shRNA (MOI = 5) for single knockdown and a mixture of two targeted gene shRNAs (MOI = 5 for each one) for double knockdown, to maintain a MOI = 10 in all conditions. P values<0.01 (**) or <0.001 (***) are indicated. (TIF) [file ppat.1003416.s008.tif]

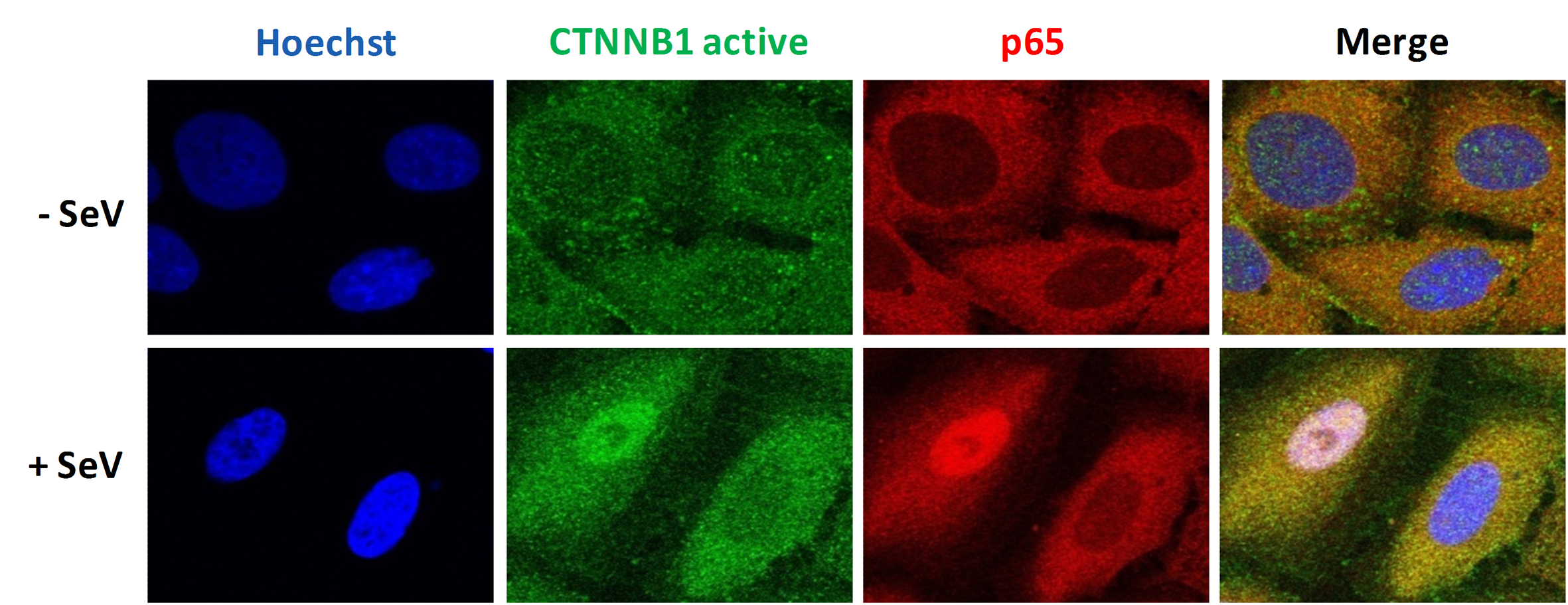

Supplement: Figure S9 — SeV infection induces CTNNB1 nuclear translocation. Confocal analysis of A549 cells using Hoechst, anti-CTNNB1 active form and anti-p65 antibodies without virus infection or following 6 hours infection with SeV. (TIF) [file ppat.1003416.s009.tif]

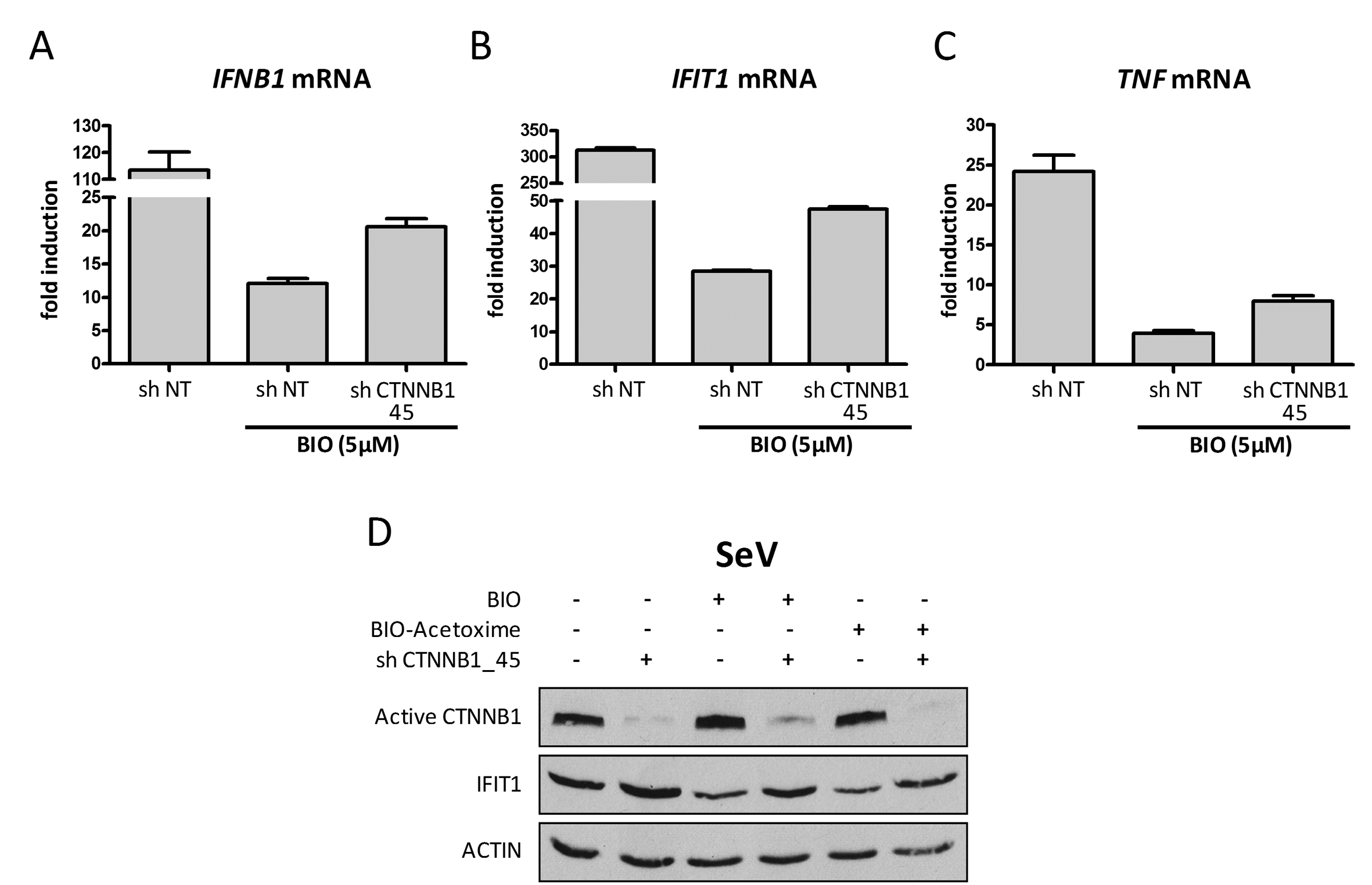

Supplement: Figure S10 — Effect of GSK3 inhibition on transcriptional activity of representative genes of innate immunity in CTNNB1 knockdown HEK 293T cells - Immunoblot analysis of CTNNB1 knockdown and GSK3 inhibitor-treated A549 cells. (A–C) qRT-PCR quantification of IFNB1 (A), IFIT1 (B) and TNF (C) mRNA levels in HEK 293T transduced with lentivirus-expressing shRNA NT (control) or shRNA 45 targeting CTNNB1 for four days and subjected to SeV infection and treatment with GSK3 inhibitor BIO (5 µM) for 16 hours. qRT-PCR determination represents the average mRNA RQ normalized versus ACTIN and HPRT1 mRNA. (D) Immunoblot analysis of dephosphorylated active CTNNB1 at Ser37/Thr41 and IFIT1 in A549 cells transduced with lentivirus-expressing shRNA NT (control) or shRNA 45 targeting CTNNB1 for four days and subjected to treatment with GSK3 inhibitors BIO (5 µM) or BIO-acetoxime (10 µM) and SeV infection for 6 hours. (TIF) [file ppat.1003416.s010.tif]

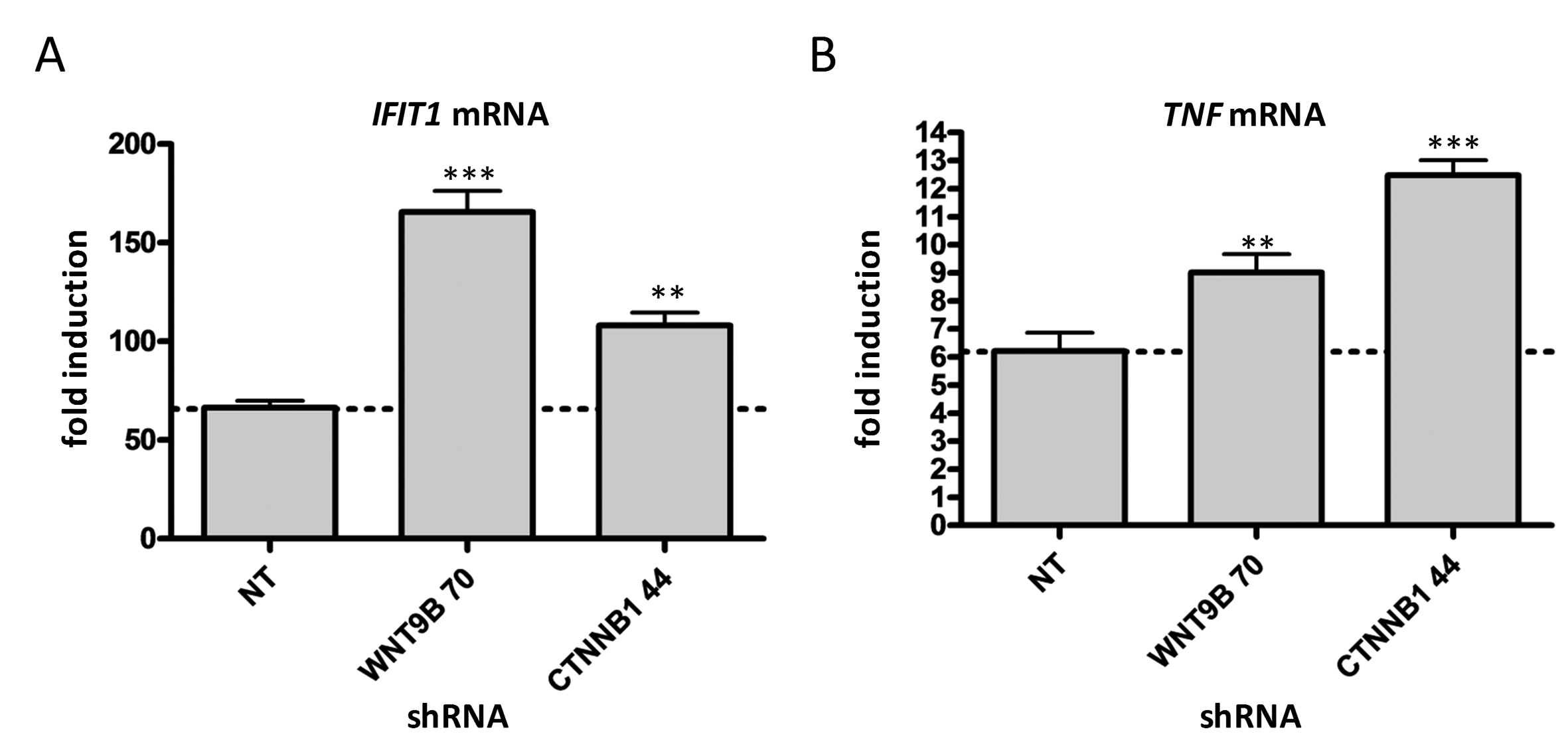

Supplement: Figure S11 — WNT/GSK3/CTNNB1 signaling acts as a negative regulator of innate immune response in primary human hepatocytes infected with SeV. Fold induction of IFIT1 (A) and TNF (B) mRNAs in primary human hepatocytes transduced with lentivirus-expressing shRNA NT, shRNA WNT9B 70 or shRNA CTNNB1 44 for four days and subjected SeV infection for 5 hours. qRT-PCR determination represents the average mRNA RQ normalized versus ACTIN and HPRT1 mRNA. P values<0.01 (**) or <0.001 (***) are indicated. (TIF) [file ppat.1003416.s011.tif]

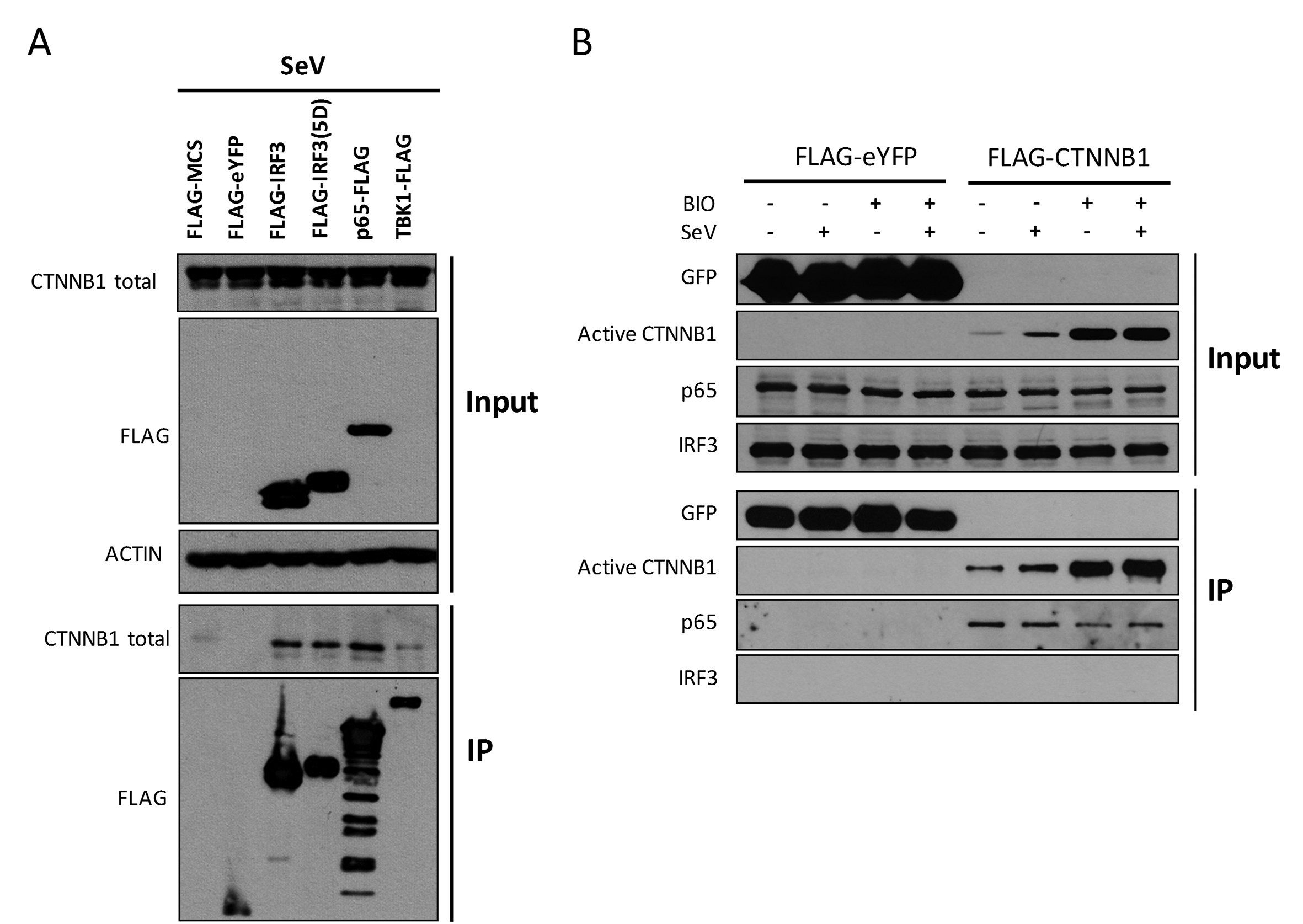

Supplement: Figure S12 — CTNNB1 associates with IRF3 and NF-κB subunit p65. (A) HEK 293T cells were co-transfected with CTNNB1 and FLAG-MCS (control), FLAG-eYFP (control), FLAG-IRF3, FLAG-IRF3(5D) p65-FLAG or TBK1-FLAG expressing plasmids for forty-eight hours. Cell extracts were prepared following infection with SeV for 16 hours before being subjected to immunoprecipitation directed against FLAG. Immune complexes were analyzed by Western blotting using anti-FLAG and anti-CTNNB1 antibodies. (B) HEK 293T cells were transfected with FLAG-eYFP (control) or FLAG-CTNNB1 expressing plasmids for forty-eight hours. Cell extracts were prepared following 16 hours of treatment with GSK3 inhibitor BIO (5 µM), SeV infection or both before being subjected to immunoprecipitation directed against FLAG. Immune complexes were analyzed by Western blotting using anti-FLAG and anti-p65 and anti-IRF3 antibodies. (TIF) [file ppat.1003416.s012.tif]
